# Integration of physiological and transcriptomic analyses regarding the effects of exogenous salicylic acid on drought resistance in *Cinnamomum camphora*

**DOI:** 10.3389/fpls.2025.1634592

**Published:** 2025-09-10

**Authors:** Tangjie Zhao, Xin Guan, Huanxian Guo, Chengbo Peng, Heng Wang, Yunbin Zhou, Tingwen He, Siting Yu, Zhu Gao, Yuan Zheng

**Affiliations:** ^1^ The Key Laboratory of Forest Resources Conservation and Utilization in the Southwest Mountains of China Ministry of Education, Southwest Forestry University, Kunming, China; ^2^ Jiangxi Provincial Key Laboratory of Plantation and High Valued Utilization of Specialty Fruit Tree and Tea, Institute of Biological Resources, Jiangxi Academy of Sciences, Nanchang, China; ^3^ College of Biological and Food Engineering, Southwest Forestry University, Kunming, China

**Keywords:** salicylic acid, drought resistance, signal transduction, sugar metabolism, phenylpropanoid

## Abstract

Salicylic acid (SA) serves as an intercellular signaling molecule, playing a crucial role in plant growth and development, along with the response to environmental stressors. However, molecular regulations that govern salicylic acid-induced resistance to drought in plants remain incompletely elucidated. This research utilized two-year-old *C. camphora* seedlings as the experimental subjects, employing a two-factor experimental design that incorporated soil moisture×salicylic acid spraying. Through a combination of physiological and transcriptomic analyses, it aimed to elucidate the mechanisms by which exogenous salicylic acid influences the growth and physiological traits of *C. camphora* seedlings subjected to drought stress, as well as the regulation of salicylic acid-mediated drought-related signaling pathways. Research indicates that SA can markedly improve the substance called chlorophyll fluorescence parameters (that is, Fv/Fm and PI_abs_) of *C. camphora* subjected to drought stress, augment photosystem activity during mild drought conditions, and mitigate the damage inflicted by excessive light energy in photosynthetic institutions. SA significantly alleviated oxidative stress in *C. camphora* seedlings under drought stress by reducing O_2_^-^ and H_2_O_2_ contents and enhancing SOD, POD, and CAT activities. Transcriptome analysis revealed that SA induces DEGs associated with drought resistance. It activates transcription factors that are attached as NAC, bHLH, ERF, and MYB, and regulates genes involved in plant hormone signaling, such as AUX/IAA, PYR/PYL, A-ARRs, and B-ARRs. Additionally, it suppresses the degradation of starch, enhances the expression of genes associated with photosynthesis, and alleviates the adverse effects during conditions of drought that negatively impact the photosynthetic performance of *C. camphora*, thus enhancing their resilience to drought conditions. Furthermore, SA significantly affected phenylpropanoid synthesis-related genes (such as CcHCT, CcPOD, and CcCOMT). This research seeks to improve understanding of the mechanisms by which SA influences drought tolerance in plants, providing novel insights into enhancing drought resistance in *C. camphora*.

## 1 Introduction

The intensification of global climate change has led to an increased frequency of extreme weather events, which presents a significant challenge to ecosystem equilibrium and functionality. Drought is one of the most common climatic extremes on a global scale and one of the most serious challenges facing humankind today (Lan et al., 2024; Sato et al., 2024). In Southwest China, particularly in the Yunnan-Guizhou Plateau and Chongqing, harsh droughts throughout spring and summer have occurred often in recent years, significantly affecting local vegetation growth and ecosystem health (Song et al., 2019). The southwestern region, recognized as China’s primary carbon sink, faces limitations due to its karst landscape, characterized by low soil water retention and vegetation susceptible to drought. This alteration endangers the local ecosystem as well as significantly influences the geographical distribution of flora, leading to a minimal survival rate of adaptable species, which impedes their capacity to absorb water and nutrients, disrupts their metabolic processes, and consequently affects their growth rate, biomass, reproductive strategies, competition, and symbiotic relationships (Zhang et al., 2021, 2025b). Therefore, employing various practical technical approaches and methods to enhance plant drought resistance and develop effective ecological conservation and adaptation strategies is crucial.

Plants facing extreme adversity, such as drought, typically exhibit several adaptive responses. These include deep rooting, leaf curvature, reduced leaf area, decreased transpiration, stomatal closure, accumulation of osmoregulatory substances, and activation of genes involved in the stress response (Tiwari et al., 2017; Zailaa et al., 2024). Nevertheless, several plant species or genotypes within a species frequently exhibit significant disparities in their sensitivity and tolerance to drought stress (Fang and Xiong, 2015). Research indicates that vegetation sensitivity to arid environments is influenced not only by its drought tolerance but also by the duration and intensity of soil moisture deficit (Liu et al., 2024). To adapt to arid environments, plants have developed three typical survival strategies over long periods of evolution: drought escape (DE), drought avoidance (DA), and drought tolerance (DT) (Fang and Xiong, 2015; Liu et al., 2024). DE refers to the natural or artificial regulation of plant phenology (such as flowering and fruiting in advance) to escape the drought period (Manavalan et al., 2009). DT involves the maintenance of cell expansion pressure through the increase of intracellular osmoregulators, thereby enhancing plant adaptation to drought conditions. Conversely, DA is a method that boosts water uptake efficiency and minimizes transpirational losses to achieve drought tolerance (Rahman et al., 2022). A complicated characteristic of plants is drought tolerance, which involves molecular, physiological, and morphological alterations. Consequently, it is essential to investigate the mechanisms underlying plant drought resistance through the lens of growth physiology and gene regulation.

Salicylic acid (SA), a phenolic compound found in plants, is important to the regulation of plant growth and development, as well as the response to environmental stressors. Its biosynthesis primarily occurs through the phenylalanine ammonia-lyase (PAL) and isochorismate synthase (ICS) pathway. Numerous investigations have demonstrated that SA, functioning as a plant growth regulator, engages with other signaling molecules and phytohormones. This interaction not only facilitates plant photosynthesis and growth but also subsequently strengthens the antioxidant system. Consequently, this mechanism effectively safeguards the plant against oxidative damage, membrane impairment, and metabolic disorders by scavenging reactive ROS and enhancing the activity of antioxidant enzymes (Li et al., 2023; Shi et al., 2024a; Torun et al., 2024). It can also significantly mitigate stress-induced damage to photosynthesis and chloroplasts, thereby enhancing plant tolerance to abiotic stresses (Cheng et al., 2020). However, depending on the species, type of stress, and environmental factors, the mechanism of SA interactions with abiotic stresses in plants varies (Torun et al., 2024). Consequently, comprehending the function of SA in the response of woody plants to abiotic stress is crucial for formulating strategies aimed at enhancing their resilience and productivity amid environmental challenges. While much research has been conducted on SA regulation of plant resistance, there are fewer reported studies on the mining of various pathways and their integration and analysis of SA regulation of drought resistance in plants.

*Cinnamomum camphora* is classified within the genus *Cinnamomum* of the Lauraceae family, which is primarily found in the southern and southwestern regions of the Yangtze River in China. As an excellent multi-purpose tree species, it is often used as a street tree, shade tree, and in scenic forests (Zhou and Yan, 2016). The plant holds considerable importance in both ecological and economic domains, as it integrates spice, medicinal, and timber applications, and is extensively utilized in industrial production and pharmaceutical manufacturing (Lee et al., 2022). Studying indicates that all components of the plant, including roots, stems, leaves, and seeds, are abundant in essential oil constituents, which exhibit anti-fungal, anti-inflammatory, anti-oxidant, and various other biological activities (Poudel et al., 2021; Huang et al., 2024). In southwestern China, seasonal droughts such as spring drought, summer drought, and mid-summer drought occur frequently, with increasing frequency of consecutive droughts during winter and spring (Song et al., 2019). The uneven distribution of water resources significantly affects the growth of *C. camphora* seedlings in subtropical regions. *C. camphora*, a significant ecological and economic tree species prevalent in the subtropical regions of southern and southwestern China, exhibits notable drought resistance to seasonal droughts. Nevertheless, the molecular physiological mechanisms by which *C. camphora* responds to drought need further investigation.

This research examined *C. camphora* seedlings, utilizing drought stress and SA application methods to assess and compare the activity of antioxidant enzymes, the production rate of reactive oxygen species, and the properties of photosynthetic fluorescence in these seedlings. Based on this foundation, the transcriptional regulatory mechanism underlying SA-induced drought tolerance was thoroughly investigated. The research primarily concentrated on the signal transduction pathways related to photosynthesis, sugar metabolism, and phenylpropanoid biosynthesis in order to clarify the molecular physiological regulatory mechanisms through which exogenous SA affects the drought tolerance of *C. camphora*.

## 2 Materials and methods

### 2.1 Test materials and design

The experimental site is situated within the greenhouse of the Arboretum at Southwest Forestry University in Kunming City, Yunnan Province, China (25°03′N, 102°45′E), positioned in a subtropical highland monsoon climate zone at an elevation of 1,964 meters.” Throughout the trial, the greenhouse received sufficient sunlight, with an average daily temperature of 20.0–31.0 °C and an average relative humidity of 22.3–48.0%. Two-year-old *C. camphora* seedlings were selected as experimental materials, with plant heights ranging from 85 to 100 cm and ground diameters from 0.7 to 1 cm. The pots used measured 21.5 cm in upper diameter, 16 cm in bottom diameter, and 20 cm in height. Each pot was filled with 2 kg of red soil (with a bulk density of 1.3 g/cm³), planted with one seedling, and subjected to regular water and fertilizer management. The soil’s saturated water content was measured at 26.58% utilizing the ring knife method (Ma et al., 2025).

A pot culture technique was employed to replicate drought conditions. In May 2023, seedlings exhibiting uniform growth were chosen for drought stress and salicylic acid spray treatment. The spray treatments comprised 50 μM salicylic acid (SA) and distilled water, while three levels of water treatments were established: suitable moisture (CK), mild drought (D1), and severe drought (D2). The soil moisture content for each treatment was 75–80%, 50–55%, and 25–30% of the saturated water-holding capacity, with actual moisture contents continued at 19.94–21.26%, 13.29–14.62%, and 6.65–7.97%, respectively. The experiment had six treatments, designated as CK, CKS, D1, D1S, D2, and D2S, respectively. A total of 72 plants were selected for the experiment, with twelve plants designated for each treatment. The soil moisture content was measured daily during the experiment using a ProCheck soil moisture meter (DECAGON, USA) (Zhang et al., 2022). Moisture was regulated by the weighing method, with pots weighed daily at 17:00 and water administered based on the weight of each treatment. Upon achieving the moisture gradient after each treatment, 5 mL was applied to both surfaces of each plant’s leaves. Spraying occurred every three days, totaling ten applications over a period of thirty days. Samples were obtained on days 1, 3, 7, 15, and 30 post-treatment, with three plants randomly chosen from each treatment group for every sampling occasion.

### 2.2 Measuring chlorophyll fluorescence parameters

During leaf collection from *C. camphora* in each treatment on days 1, 3, 7, 15, and 30 after the experimental treatment, *in situ* chlorophyll fluorescence determinations were performed from 9 to 11 am. For each treatment, three healthy camphor trees free from pests and diseases were selected. From each plant, the 5th-7th healthy leaves with good growth in the middle-upper part were taken and marked. Before the determination, they were fully light-adapted and then dark-treated for 30 minutes, and the determination was repeated three times. Fluorescent parameters, including initial fluorescent intensity (F0), maximum fluorescent intensity (FM), maximum photochemical efficiency (Fv/FM), and photosynthetic performance indicator (PI_abs_), were evaluated using the high-speed continuous excitation fluorometer, PEA-Plus (Hansatech, UK).

### 2.3 Measurement of antioxidant enzyme activities

Initially, 0.5 g of the sample was accurately measured and transferred to a pre-cooled mortar, where it was meticulously ground under liquid nitrogen until it achieved a fine powder consistency. Following this, tissue homogenization was conducted through the addition of 50 mM phosphate buffer at a pH of 7.8. The homogenate obtained was subjected to centrifugation at 10,000 r/min for a duration of 15 minutes at a temperature of 4°C. The supernatant was then collected for further analysis in the quantitative assessment of antioxidant enzyme activity.

The inhibition of nitroblue tetrazolium (NBT) reduction by superoxide dismutase (SOD) was employed to assess its activity at 560 nm, following the methodology outlined by Shu et al. (2023), with minor adjustments made to the method. Peroxidase (POD) enzyme activity was measured by the guaiacolytic method, while catalase (CAT) activity was measured at 240 nm wavelength.

### 2.4 Testing of hydrogen peroxide (H_2_O_2_) and superoxide radical (O_2_^-^) levels

The content of H_2_O_2_ was assessed by initially weighing 0.5 g within a fresh sample and subsequently incorporating 1.5 mL taken from a 0.1% TCA solution while maintaining it together under ice bath conditions for the grinding process. The homogenate underwent centrifugation at 3000 revolutions per minute for a duration of 10 minutes at a temperature of 4°C. Following this, 0.5 mL of that supernatant was aliquoted and mixed with an equivalent volume of potassium dihydrogen phosphate buffer, in combination with 1 mL of KI solution, and allowed to react for a duration of 60 minutes. The determination of superoxide anion (O_2_^-^) content was conducted utilizing the methodology outlined by Zhang et al. (2023a), with certain modifications implemented.

### 2.5 RNA extraction and transcriptome sequence analysis

On the 7th day of moisture treatment, mature leaves from the same leaf position in the upper middle of the standard plant for each treatment were collected, utilizing three biological replicates per treatment. The samples were subsequently numbered after mixing the samples from each treatment separately (CK_1, CK_2, CK_3, CKS_1, CKS_2, CKS_3, D1_1, D1_2, D1_3, D1S_1, D1S_2, D1S_3, D2_1, D2_2, D2_3, D2S_1, D2S_2, and D2S_3). A total of 18 samples were cryopreserved using dry ice and subsequently sent to Suzhou PanoMicro Biomedical Technology Co., Ltd. The Illumina Plant RNA Extraction Kit (San Diego, CA, USA) was used to extract total RNA from 18 C*. camphora* leaf samples. The assessment of the concentration and purity of the extracted RNA was conducted using a Thermo Scientific NanoDrop 2000 instrument for the analysis. The assessment of RNA integrity was conducted utilizing agarose gel electrophoresis techniques specifically designed for RNA, or through the application of Agilent’s 2100 Bioanalyzer (with RNA 6000 nano kit 5067-1511).

To ensure comparability of expression data across samples and genes, transcriptome data were normalized using the DESeq analysis toolkit in R, and principal component analysis (PCA) was conducted based on the normalized expression matrices to evaluate the overall pattern of differences between samples. The DESeq tool was employed for the detection of differential gene expression (DEGs), with screening criteria established as follows: a magnitude in fold change |log2FoldChange|>1, while a statistical significance threshold of P <0.05. DEGs were subjected to enrichment analysis utilizing the Gene Ontology (GO) and Kyoto Encyclopedia of Genes and Genomes (KEGG) databases, with a significance threshold set at P <0.05, indicating notable enrichment results.

### 2.6 Quantification of fluorescence in real time

For the purpose of assessing the reliability that was RNA-seq findings, eight genes were selected at random and analyzed using qRT-PCR methodology. The design of the primers was carried out using the Primer Premier 6.0 software, and the corresponding primer sequences are shown in **Supplementary Table S1**. Reverse transcription was conducted utilizing the FastKing RT Kit (KR116) reagent. The reaction system comprised 10 μL of Power qPCR PreMix (Genecopoeia), 0.5 μL Primer_F, 0.5 μL Primer_R, 1 μL cDNA, and 8 μL H2O, totaling 20 μL. The reaction parameters consist of an initial pre-denaturation step at 95°C for 15 minutes, followed by denaturation at 95°C for 10 seconds, and subsequent annealing and extension at 60°C for 40 seconds, culminating in a total of 40 cycles. Determination of relative gene expression employing the 2-^ΔΔCt^ methodology.

### 2.7 Statistical analyses

Data analysis was conducted utilizing Microsoft Excel 2017 and SPSS 24.0 to perform significance testing and Pearson correlation analysis. Additionally, Statistical analyses were conducted utilizing Two-Way ANOVA, with a significance threshold set at P < 0.05 to determine statistically significant differences. Graph construction utilized GraphPad Prism 9 software, while metabolic heat mapping was performed using TBtools. Analysis of KEGG pathway and GO function annotation for differentially expressed genes was conducted utilizing the Chiplot online platform (https://www.chiplot.online/) alongside the Microbiology Letter website (https://www.bioinformatics.com.cn/).

## 3 Results and analysis

### 3.1 Drought stress and exogenous SA influence on the phenotypic traits of *C. camphora*

**Figure 1:**
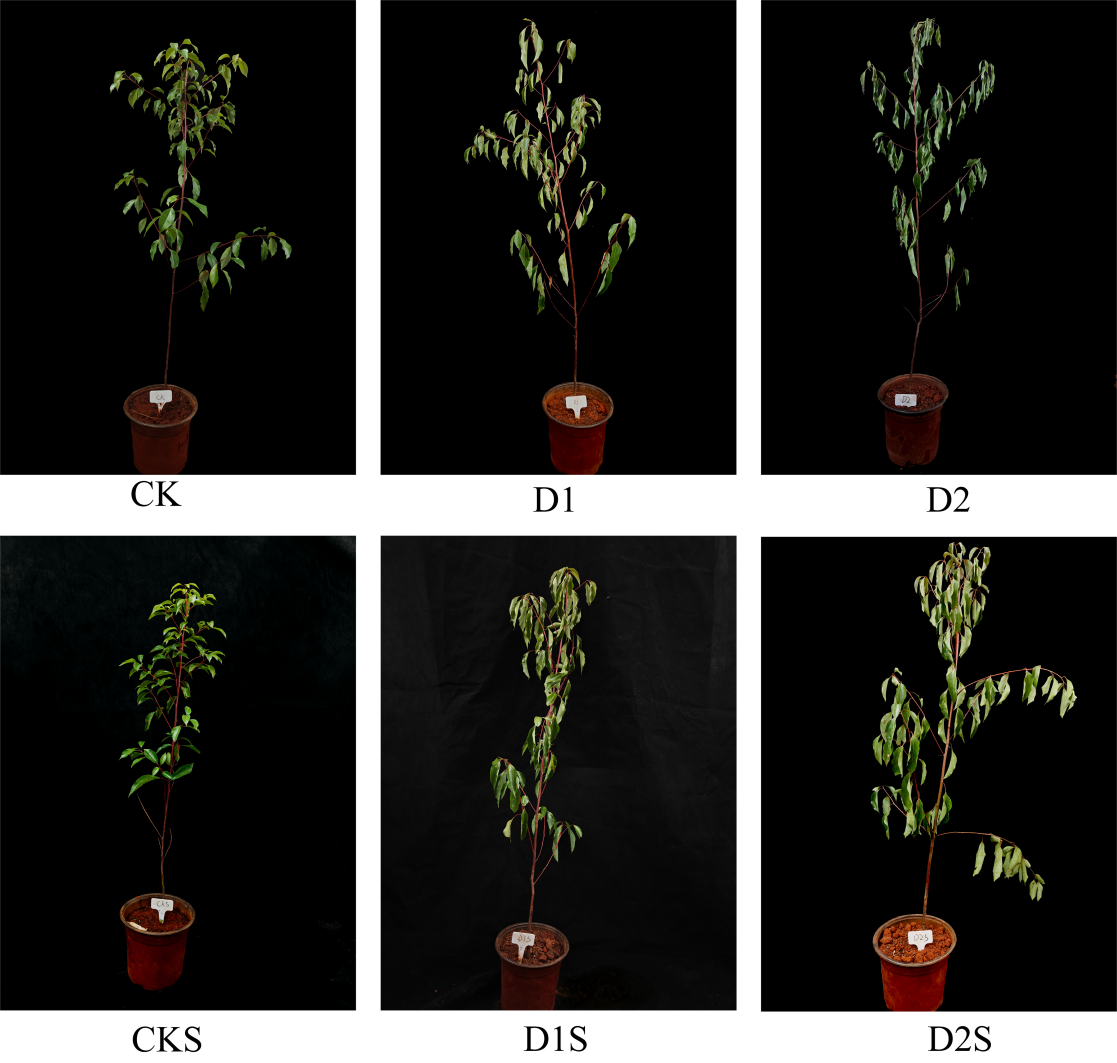
Effects on *C. camphora* phenotypes of drought stress and exogenous SA.

To investigate the phenotypic alterations of *C. camphora* under drought stress and exogenous SA treatment, we captured images of their stress phenotypes across several treatments. Drought stress negatively impacts the growth of *C. camphora* by decreasing soil water content, resulting in water loss in cellular tissues, which is obvious in leaf chlorosis and loss of greenness (**Figure 1**). As drought stress intensified, severe stress led to curling of the entire plant leaves, and the chlorosis of *C. camphora* leaves was notably mitigated by the use of exogenous SA. This suggests that exogenous SA alleviated the impacts of drought stress on *C. camphora* to some degree, particularly under mild drought conditions, thereby supporting the growth of the plant.

### 3.2 Effects on chlorophyll fluorescence parameters of drought stress and exogenous SA

**Figure 2:**
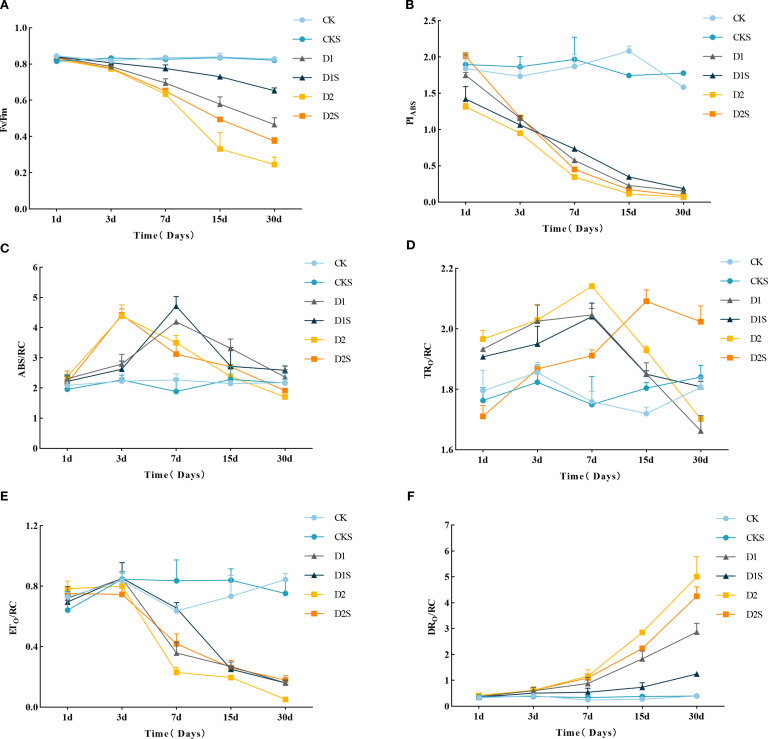
Six-treatment *C. camphora* seedling photosynthetic comparison. **(A)** Maximum photochemical efficiency (Fv/Fm), **(B)** Absorption-based performance index (PI abs), **(C)** Light energy absorbed per unit of reaction center (ABS/RC), **(D)** Light energy captured per unit of reaction center (TRo/RC), **(E)** Energy utilized per unit of reaction center for electron transport (ETo/RC), **(F)** Energy dissipated per unit of reaction center as heat (DIo/RC). Different lowercase letters indicate that differences are statistically significant at the p < 0.05 level.

**Figure 2** shows that as drought stress duration increases, Fv/Fm (**Figure 2A**) and PI_ABS_ (**Figure 2B**) fall, with significant differences between treatments. Fv/Fm and PIABS decreased in D1 and D2 after three days of drought treatment. After 30 days, Fv/Fm decreased by 43.37% and 69.88% (P<0.05) compared to the control, while PI_abs_ decreased by 90.51% and 95.57% (P<0.05), indicating that PI_ABS_ exhibited greater sensitivity to drought conditions. Exogenous SA increased Fv/Fm by 38.30% and 52.00% (P < 0.05) and PI_abs_ by 26.67% and 28.57% (P < 0.05) compared to D1 and D2. The results show that drought stress may degrade the PSII system, whereas exogenous SA protects the photosynthetic system and increases *C. camphora* photosynthetic capacity under drought circumstances.

Furthermore, as drought stress escalated, alterations in the parameters of PSII response center activity were observed (**Figures 2C–F**). The ABS/RC initially rose and subsequently declined with extended stress, reaching its zenith on days 3–7 and 1–3 of D1 and D2 treatments, exhibiting increases of 84.58% and 96.41% over the CK, respectively (P<0.05). Following SA treatment, D1S enhanced the elevation of ABS/RC, whereas D2S did not demonstrate a significant impact (**Figure 2C**). TRO/RC increased and then decreased under D1 and D2, peaking at day 7 with increases of 16.48% and 21.59% (P<0.05) compared to CK, respectively. D1S delayed the rate of decline in the later stage, while D2S peaked at 15 days and then declined, decreasing 8.29% (P<0.05) compared to the drought stress treatment (**Figure 2D**). Drought stress reduced ETO/RC without any significant variation among treatments (**Figure 2E**). The DIO/RC escalated with both the intensification and duration of stress, while the administration of exogenous SA significantly augmented DIO/RC by 56.79% and 15% (P<0.05) compared to D1 and D2, respectively (**Figure 2F**). It suggested that exogenous SA may diminish heat dissipation by augmenting light energy absorption (ABS/RC) and conversion (TRO/RC, ETO/RC).

### 3.3 The impact of exogenous SA on reactive oxygen species activity and antioxidant enzyme levels in *C. camphora* subjected to drought stress

**Figure 3:**
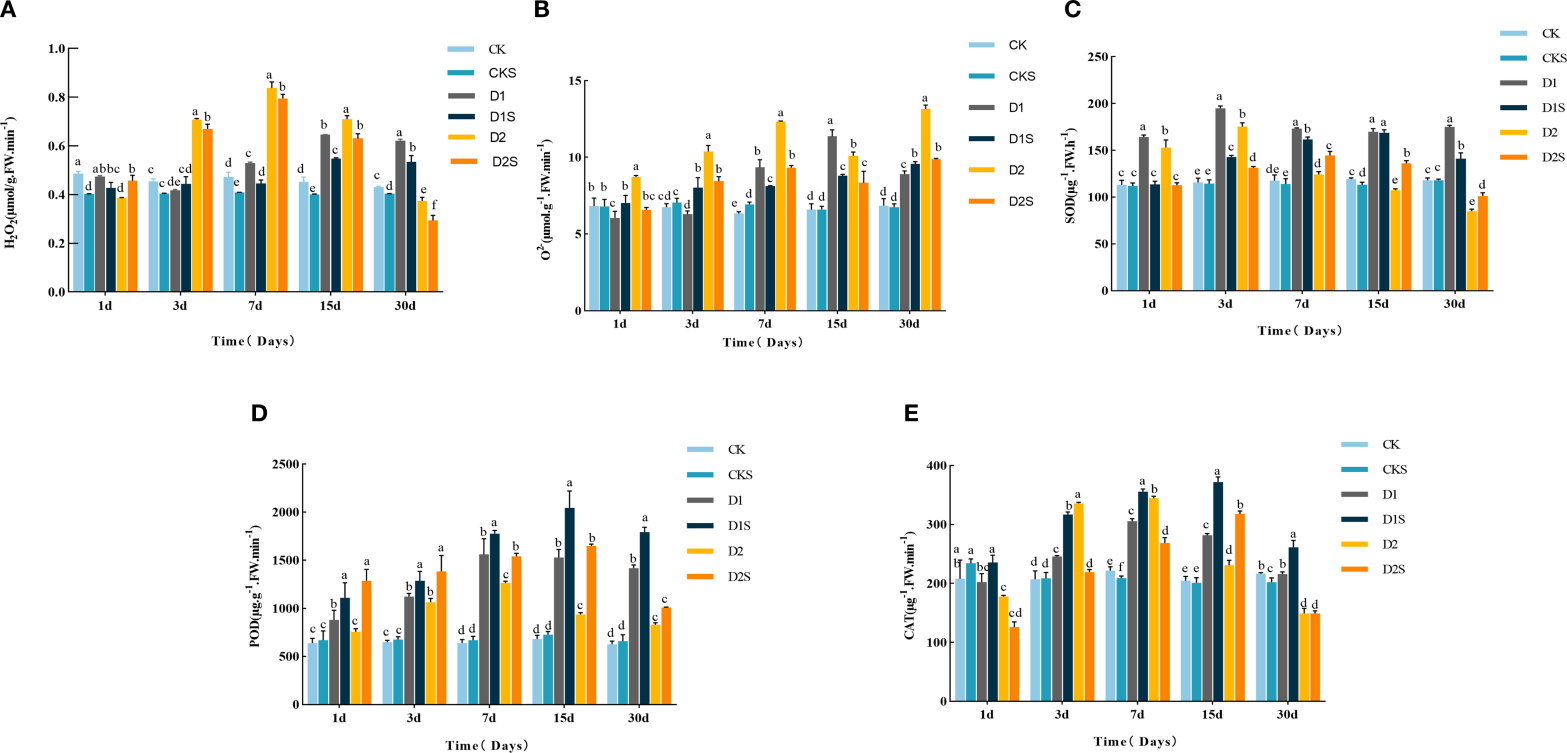
Shows drought-stressed ROS content and antioxidant enzyme activity. **(A)** Hydrogen peroxide (H2O2), **(B)** superoxide anion (O2-), **(C)** superoxide dismutase (SOD), **(D)** peroxidase (POD) activity, **(E)** catalase (CAT) activity. Different lowercase letters indicate that differences are statistically significant at the p < 0.05 level.

**Figure 3A** shows that D1 treatment increased H_2_O_2_ levels with prolonged stress duration, while D2 treatment showed a parabolic fall, peaking at 15 and 7 days, respectively, with 44.44% and 78.72% increases over CK (P<0.05). Nonetheless, the administration of SA (D1S, D2S) markedly diminished the accumulation of reactive oxygen species and lowered the H_2_O_2_ content to below its initial level. **Figure 3B** illustrates that drought stress markedly elevated O_2_^-^content (P < 0.05) with the duration of stress during the drought treatment, with increases of 47.32%, 72.27%, 29.74%, 94.64%, 52.88%, and 91.84% at 7d, 15d, and 30d in D1 and D2, respectively, when compared to CK (P < 0.05). Conversely, SA treatment significantly diminished O_2_^-^content, which decreased over time under D2S treatment, with reductions of 24.63%, 18.69%, 24.39%, 17.24%, and 25% (P < 0.05) at the 1st, 3rd, 7th, 15th, and 30th days, respectively, compared to D2 treatment. Consequently, topically administered SA can significantly mitigate oxidative damage induced by a lack of water in *C. camphora* seedlings.

Meanwhile, the SOD activity in *C. camphora* seedlings shown in **Figure 3C**, first rose and then fell with the stress time the D1 and D2 treatments peaked on day 3, significantly improved by 68.10% and 50.86% relative to CK (P<0.05). Exogenous SA-treated D1S and D2S reached their peak on days 15 and 7, respectively, with enhancements of 42.01% and 22.88% relative to D1 and D2 treatments (P<0.05). **Figure 3D** shows POD activity peaks on days 15 and 7, with D1 and D2 treatments showing 124.05% and 98.43% (P<0.05) increases compared to CK. Additionally, exogenous SA-treated D1S and D2S demonstrated 33.84% and 74.14% (P<0.05) increases compared to D1 and D2 treatments. **Figure 3E** shows that CAT activity peaked on day 7 for D1 and D2 treatments, with increases of 37.39% and 55.41% (P<0.05) compared to CK, and on day 15 for exogenous SA-treated D1S and D2S, with increases of 32.38% and 37.66% (P<0.05) compared to D1 and D2 treatments. Exogenous SA significantly augmented the leaf antioxidant enzyme activities in *C. camphora* seedlings, preserved the scavenging of ROS, and diminished the rate of ROS production, with short-term mild drought proving more effective than long-term severe drought.

### 3.4 RNA-Seq data evaluation and differential gene analysis

**Figure 4:**
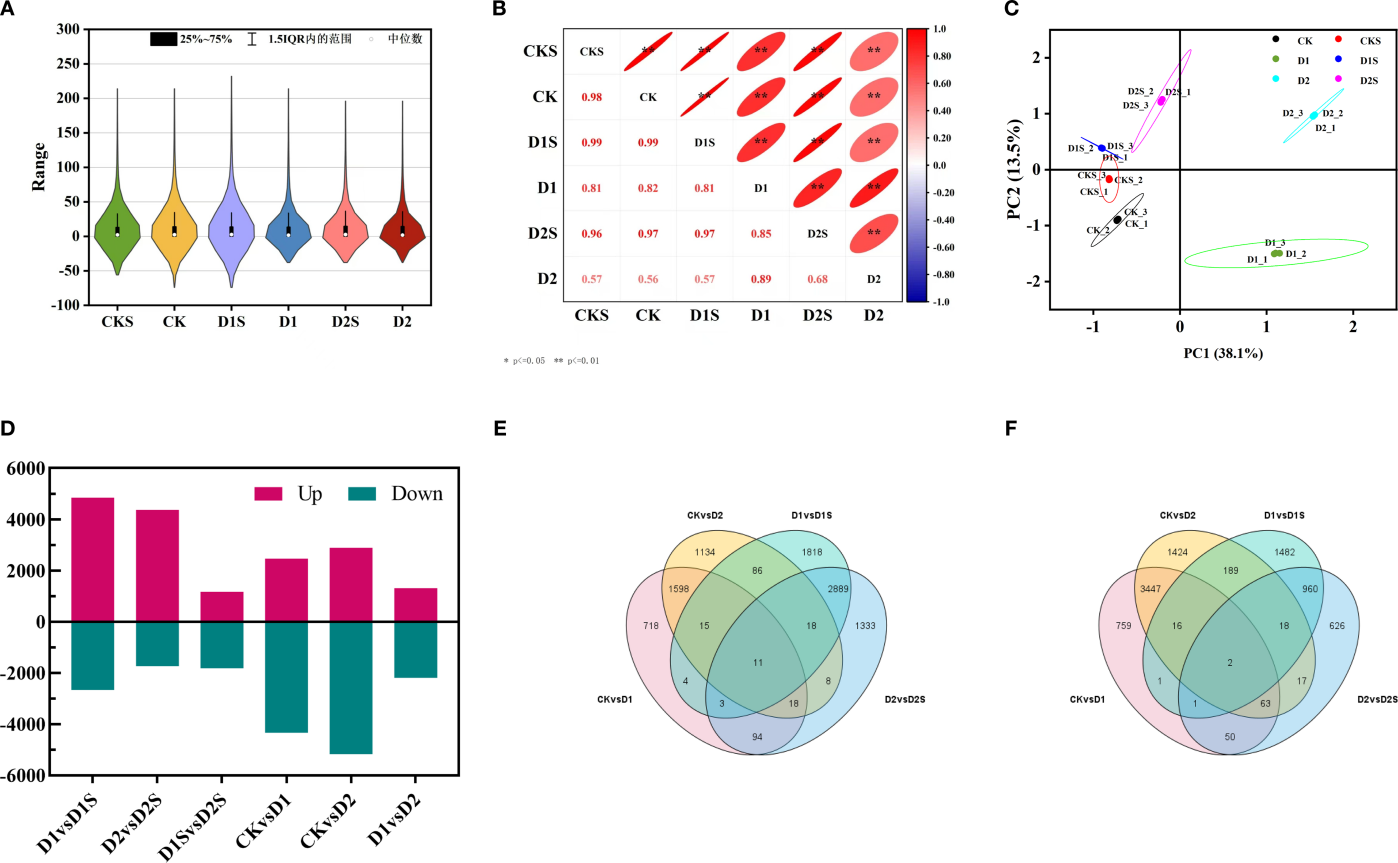
Correlation with differentially expressed genes across treatments. **(A)** Statistical outcomes of gene expression profiles in distinct samples. **(B)** Heat map illustrating the correlation among samples. **(C)** PCA was performed for each sample based on expression levels. **(D)** Expression difference results analysis. **(E)** The Venn diagram illustrates the quantity of DEGs up- and down-regulated in the four comparisons **(F)**.

This research seeks to clarify the molecular regulatory mechanisms through which exogenous SA mitigates drought stress in *C. camphora*. RNA sequencing analyses were conducted on a sample set comprising CK, CKS, D1, D1S, D2, and D2S, resulting in the construction of 18 cDNA libraries. Following the filtration of low-quality reads, the resultant clean sequencing data averaged 37 to 49 million reads per sample, with a base quality value Q30 exceeding 92.89% across all samples, thereby ensuring the reliability and accuracy of subsequent analyses. The reference genome comparison analysis indicated that the comparison efficiency of the clean reads for each sample ranged from 92.60% -93.75%. This suggests that the RNA-Seq data quality was reliable, making the obtained transcriptome sequencing data appropriate for further in-depth studies (**Supplementary Table S2**). FPKM density distribution shows that most genes in the sample are medium-expressed, with a small percentage of low and highly expressed genes (**Figure 4A**). We used the Pearson correlation coefficient to measure this correlation and found that the treatment coefficients ranged from 0.56 to 0.98, showing increased expression pattern similarity (**Figure 4B**). Notable alterations in gene expression across the three replicates of biology for each treatment have been discovered through PCA of the RNA-seq data (**Figure 4C**).

**Figure 5:**
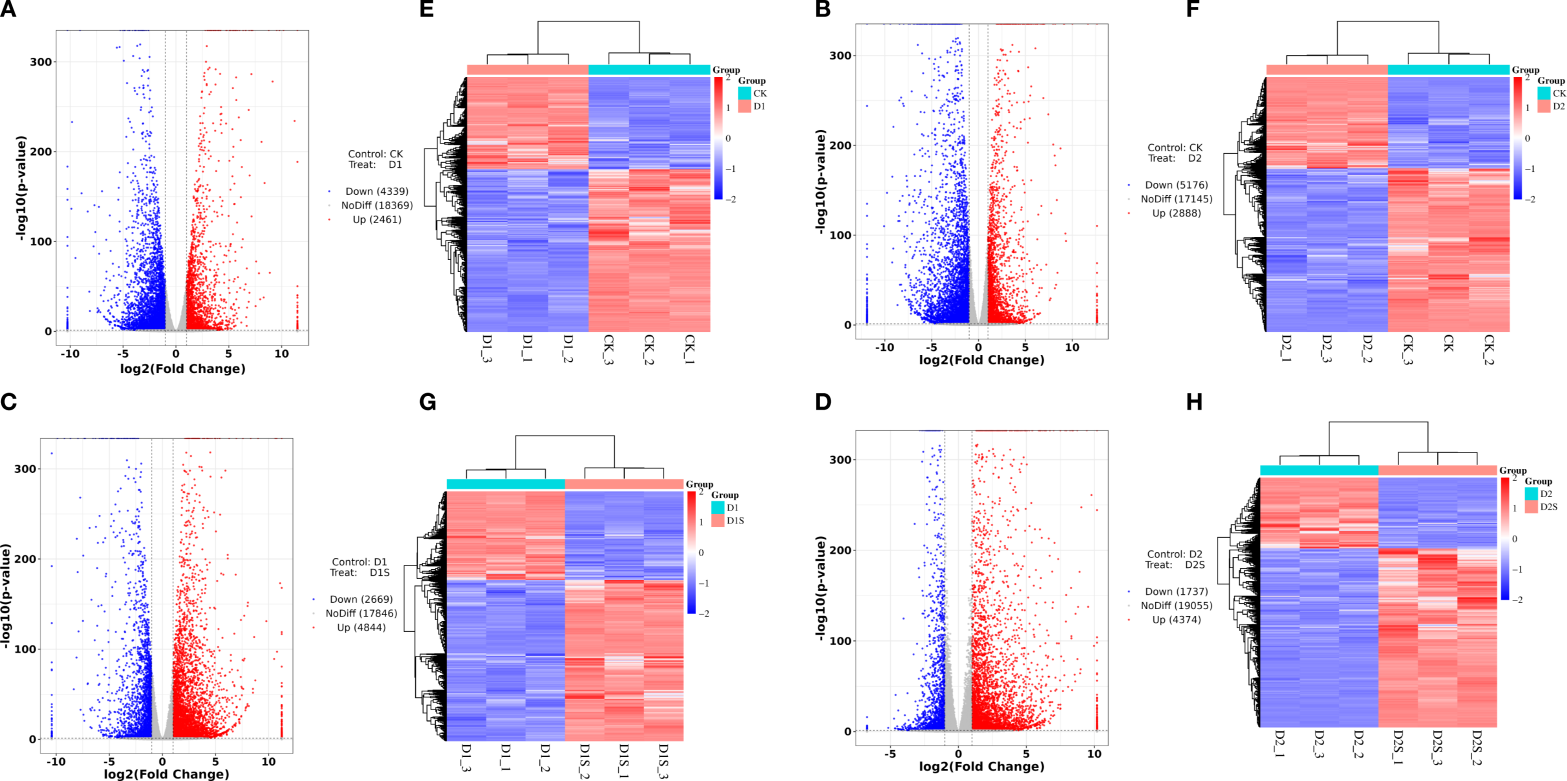
**(A–D)** Volcano plots and **(E–H)** clustered heat maps of DEGs comparing CK *vs* D1, CK *vs* D2, D1 *vs* D1S, and D2 *vs* D2S. The X-axis delineates the sample name and clustering level, while the Y-axis illustrates the DEGs and clustering groups. The volcano icon illustrates the regulation of genes, with red dots representing up-regulation, blue dots denoting down-regulation, and grey dots indicating no significant difference in gene expression. The results of the cluster analysis indicate that red signifies high expression levels, while blue denotes low expression levels.

The DEGs between the groups were further analyzed to investigate SA-induced gene expression in drought-stressed *C. camphora*. We identified DEGs with | log2 (foldchange) | > 1, P < 0.05 (**Supplementary Table S3**, **Figure 4D**). The findings revealed that 6800 DEGs were identified in CK *vs*. D1 (2461 up- and 4339 down-regulated; **Figure 5A**), 8064 DEGs in CK *vs*. D2 (2888 up- and 5176 down-regulated; **Figure 5B**), 7513 DEGs in D1 *vs*. D1S (4844 up- and 2669 down-regulated; **Figure 5C**), and 6111 DEGs in D2 *vs*. D2S (4374 up- and 1737 down-regulated; **Figure 5D**). In addition, we performed a comprehensive analysis of DEGs in CK *vs*. D1, CK *vs*. D2, D1 *vs*. D1S, and D2 *vs*. D2S, revealing a total of 13 induced transcripts (**Figures 4E, F**).

### 3.5 Annotate and enrich GO and KEGG pathways

**Figure 6:**
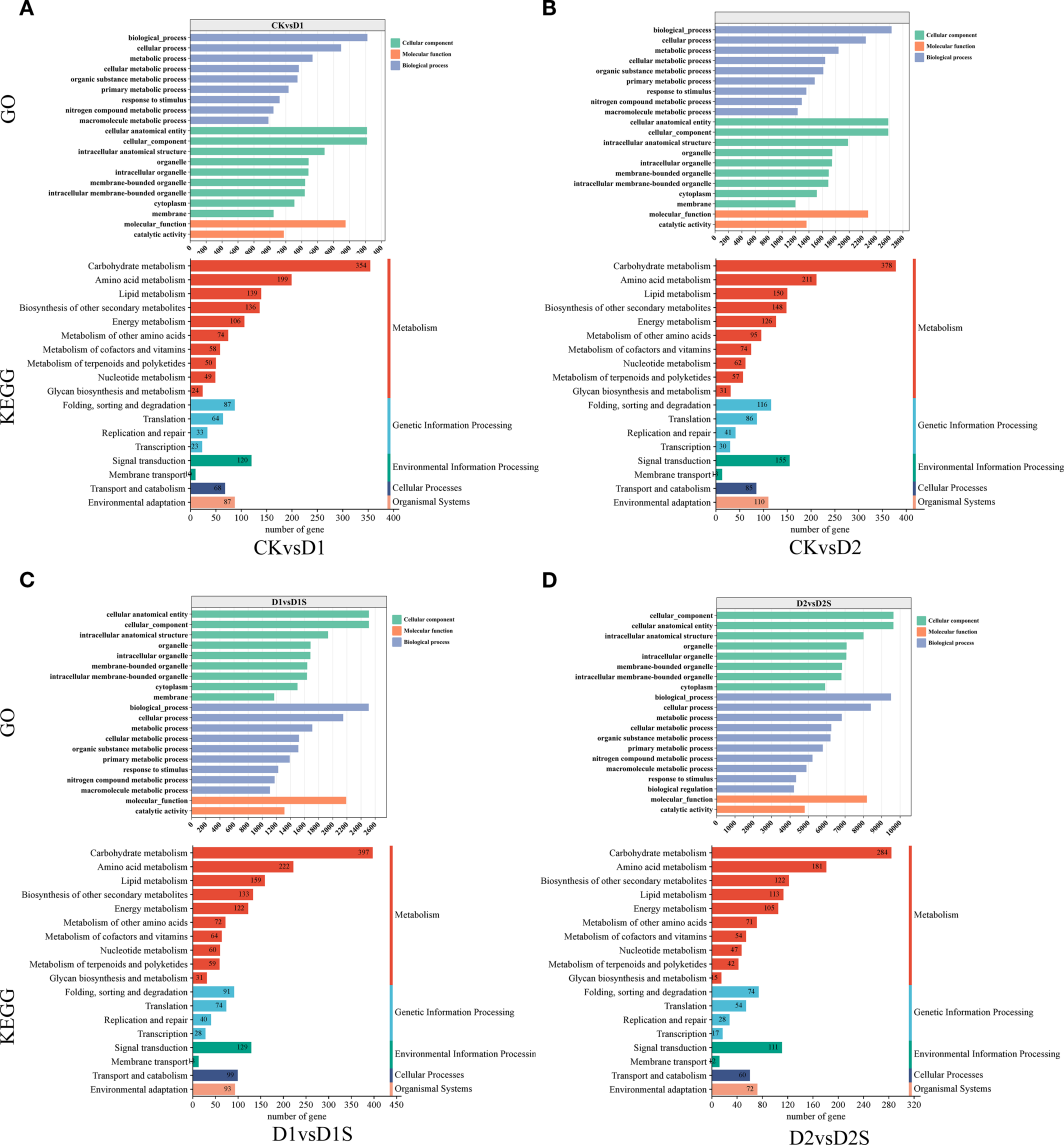
Analysis of GO and KEGG terms related to the top 20 annotated DEGs: **(A)** CK *vs* D1, **(B)** CK *vs* D2, **(C)** D1 *vs* D1S, and **(D)** D2 *vs* D2S. The x-axis represents the quantity of genes within each classification, while the y-axis denotes the GO and Kyoto Encyclopedia of KEGG classifications.

GO annotation results indicated that DEGs were grouped into three major categorizations: biological processes (BP), molecular functions (MF), and cellular components (CC). These classifications were utilized in four distinct comparative analyses. The ten GO entries exhibiting the lowest P-values or the most significant enrichment within each GO category were selected for presentation (**Figure 6**; **Supplementary Table S4**). Furthermore, KEGG pathway enrichment analysis showed significant enrichment of the DEGs for secondary metabolizing pathways. DEGs were significantly enriched for secondary metabolite biosynthesis and carbohydrate metabolism in all four comparison groups. Additionally, it exhibits a significant enrichment within the environmental information processing pathway (**Figure 6**; **Supplementary Table S5**).

**Figure 7:**
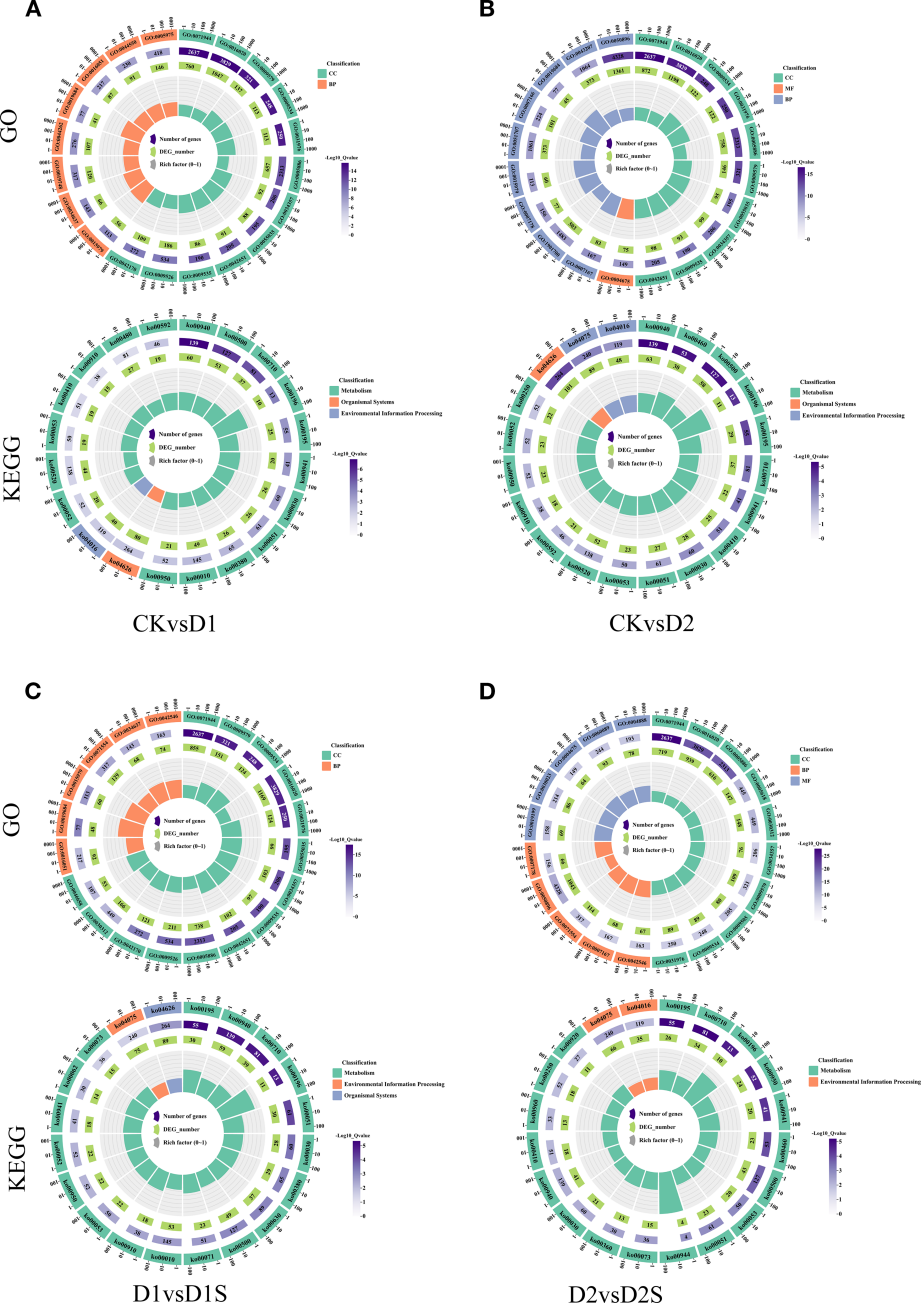
GO and KEGG pathway analyses are presented for the top 20 significantly enriched differentially expressed genes: **(A)** CK *vs* D1, **(B)** CK *vs* D2, **(C)** D1 *vs* D1S, and **(D)** D2 *vs* D2S.

Using GO and KEGG enrichment analysis, a systematic approach was used to characterize DEGs under drought stress and exogenous SA treatment. Enrichment was assessed quantitatively using the Rich factor, false discovery rate (FDR), and number of pathway-related genes. Ultimately, the 20 most significant enriched entries and metabolic pathways were selected (**Figure 7**). Comparative analyses revealed that the enrichment of terms related to the cell periphery and membrane was most significant when comparing CK *vs* D1 or CK *vs* D2 groups. The D1 *vs* D1S group exhibited significant enrichment in the cell periphery, vesicles, and chloroplast-like vesicles, while the D2 *vs* D2S group demonstrated notable enrichment in the cell periphery, membranes, and plasma membrane regions. KEGG pathway analyses indicated significant enrichment of the phenylketone analogue synthesis pathway and the metabolism of starch and sucrose in both groups (CK *vs* D1 and CK *vs* D2). In D1 *vs*. D1S and D2 *vs*. D2S, photosynthesis was the most obvious pathway of enrichment.

### 3.6 Exogenous SA affects endogenous hormone signaling pathways in *C. camphora* under drought stress

**Figure 8:**
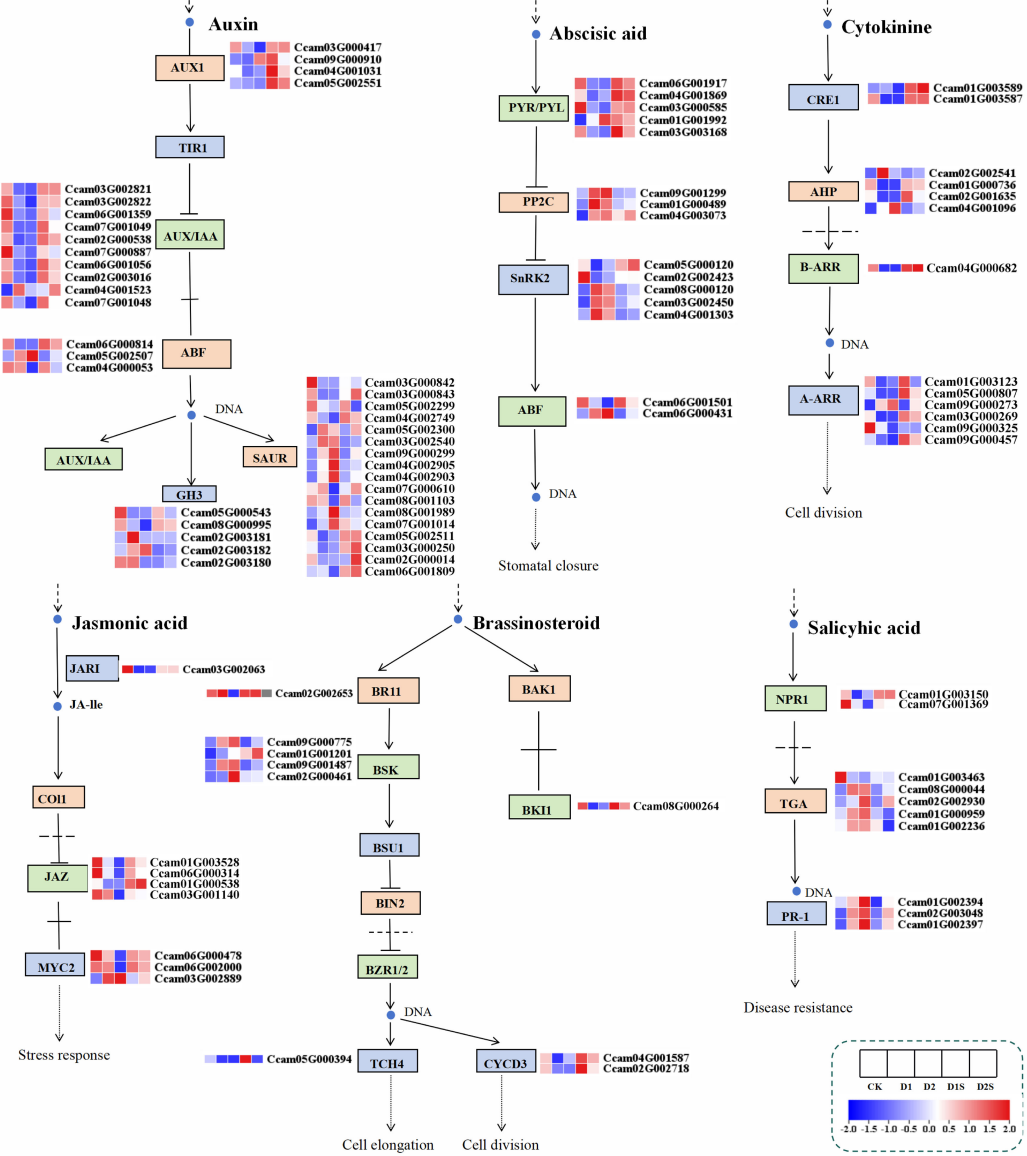
illustrates the DEGs associated with phytohormone signal transduction in response to drought stress and SA treatments. Heat map derived from FPKM of differentially expressed genes.

This research conducted a systematic analysis of the hormone signaling transduction mechanisms in *C. camphora* plants subjected to drought stress by screening DEGs within the hormone signaling pathway. The expression of eight classes of phytohormone genes was primarily analyzed, including 41 growth hormone (IAA)-related genes, 15 cytokinins (CTK), 15 abscisic acid (ABA), 6 gibberellins (GA), 8 ethylene (Eth), 8 jasmonic acid (JA), 10 oleoresinol steroids (BR), and 11 salicylic acid (SA) (**Figure 8**; **Supplementary Table S6**).

In comparisons between CK *vs* D1 and CK *vs* D2, genes linked with growth hormone response-regulating proteins (AUX/IAA) were down-regulated in the IAA production pathway. In contrast, D1 *vs* D1S and D2 *vs* D2S showed a considerable increase in their expression levels. Uniquely, genes like ARF, GH3, and SAUR exhibited a complex pattern of expression alterations in response to various treatment conditions, characterized by a combination of both up- and down-regulation. The application of SA in the ABA synthesis pathway resulted in an up-regulation of PYR/PYL receptor family gene expression and a simultaneous down-regulation of PP2C and SnRK2 gene transcript levels, relative to drought stress conditions In the CTK biosynthesis pathway, the Arabidopsis histidine kinase 2/3/4 (CRE1) gene, which functions as a cytokinin receptor, and its downstream B-Arabidopsis response-regulated transcription factors (B-ARRs) showed significantly downregulated expression in the CK *vs* D1 and CK *vs* D2 comparisons. Conversely, the comparisons D1 *vs* D1S and D2 *vs* D2S showed a significantly up-regulated expression trend. In the context of drought stress, the expression levels of JAR1, MYC2, and JAZ within the JA pathway exhibited a downregulation when comparing the CK *vs* D1. Interestingly, our analysis discovered that the expression levels of JAZ and MYC2 in the D2 versus D2S contrast exhibited an inverse trend compared to those observed in the CK versus D2 comparison.

BSK, a downstream transcription factor in the BR pathway, was up-regulated during drought stress, while BKI1, TCH4, CYCD3, and BR receptor BRI1 were down-regulated. Following SA treatment, BKI1, TCH4, CYCD3, and BRI1 exhibited significant up-regulation, whereas BSK expression displayed a combination of up- and down-regulation. Within the SA biosynthesis pathway, the PR-1 gene exhibited upregulation, whilst the NPR1 gene showed downregulation in the CK *vs* D1 and CK *vs* D2. Conversely, in the D1 *vs* D1S and D2 *vs* D2S groups, the NPR1 gene was upregulated, while the transcriptional regulators TGA and PR-1 genes were downregulated. Our research suggests that SA may play a significant role in stress adversity as well as in plant growth and development through influencing the expression of essential genes within the phytohormone metabolic pathway.

### 3.7 Exogenous SA affects the phenylpropanoid pathways in *C. camphora* during drought stress

**Figure 9:**
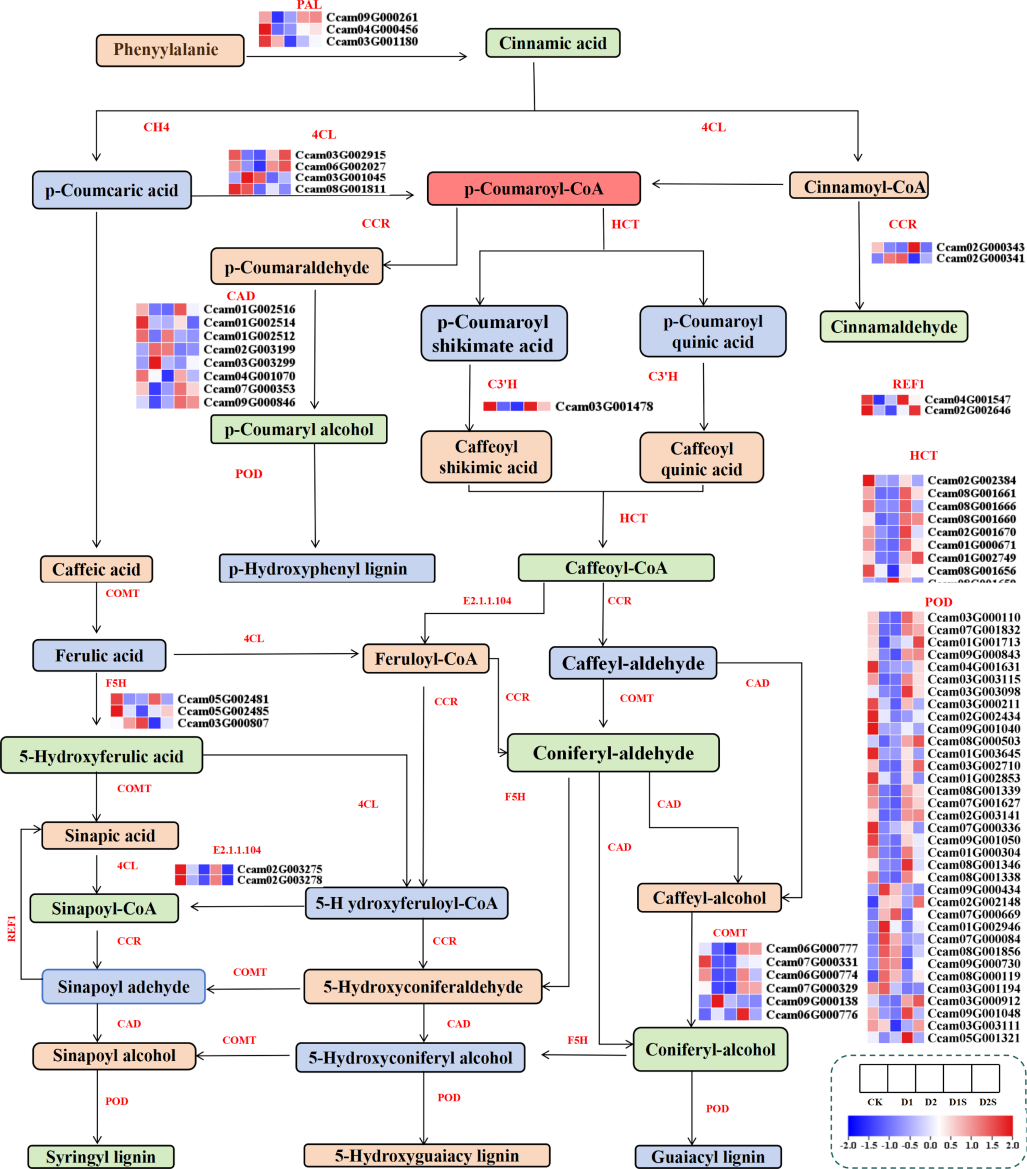
Changes in the expression of genes associated with phenylpropanoid pathways in response to drought stress and SA treatment. PAL, phenylalanine ammonia-lyase; F5H, ferulate 5-hydroxylase; COMT, caffeic acid 3-O-methyltransferase; CAD, cinnamyl-alcohol dehydrogenase; C C3’H, 5-O-(4-coumaroyl)-D-quinate 3’-monooxygenase; 4CL, 4-coumarate CoA ligase; HCT, shikimate O-hydroxycinnamoyltransferase; REF1, coniferyl aldehyde dehydrogenase; E2.1.1.104, caffeoyl-CoA O-methyltransferase; C4H, cinnamic acid 4-hydroxylase.

For research on the influence of exogenous SA on *C. camphora* phenylpropanoid, we thoroughly screened phenylpropanoid synthesis-related genes and analyzed their expression under drought circumstances (**Figure 9**; **Supplementary Table S7**). The 77 DEGS related to phenylpropanoid synthesis were screened for downregulation of PAL, HCT, F5H, REF1, and C3’H in CK*vs*D1.Whereas CCR was up-regulated, and PAL, 4CL, CAD, HCT, C3’H, F5H, and REF1 were down-regulated in CK*vs*D2. Conversely, in the D1 *vs* D1S comparison, PAL, CAD, HCT, C3’H, and REF1 were significantly upregulated, while F5H was downregulated. Additionally, in the D2 *vs* D2S comparison, PAL, POD, HCT, C3’H, COMT, and REF1 were upregulated, while F5H was downregulated.

### 3.8 The impact of SA treatment on the expression of genes associated with starch and sucrose metabolic pathways in *C. camphora* plants subjected to drought conditions

**Figure 10:**
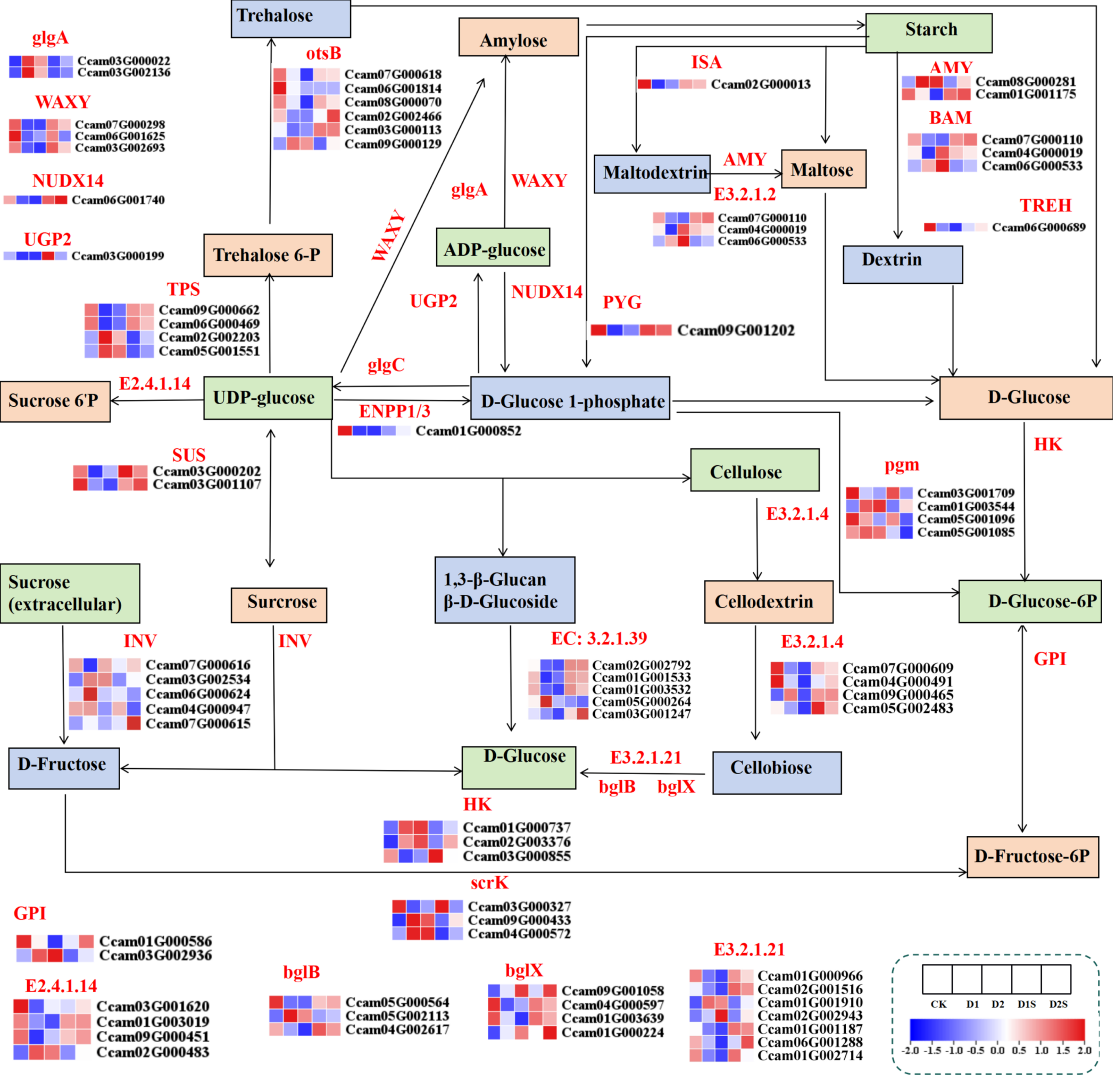
Shows the impact of drought stress and exogenous SA treatment on the expression of different genes associated with starch and sucrose metabolism in plants. FPKM heatmap of differential genes. HK, hexokinase; otsB, trehalose-6-phosphate phosphatase; AMY, alpha-amylase; E 3.2.1.21, β-glucosidase; TREH, alpha,alpha-trehalase; glgC, glucose-1-phosphate adenylyl transferase; TPS, trehalose-6-phosphate synthase; E 3.2.1.4, endoglucanase; PYG, glycogen phosphorylase; BAM (E 3.2.1.2), beta-amylase; pgm, phosphoglucomutase; glgA (EC: 2.4.1.21), starch synthase; GPI, glucose-6-phosphate isomerase; WAXY, granule-bound starch synthase; NUDX14, ADP-sugar diphosphatase; AMY, α-amylase; E 3.2.1.39, glucan endo-1,3-beta-D-glucosidase.

The research revealed that water stress had a significant impact on the expression of genes associated with starch and sucrose metabolic pathways (**Figure 10**; **Supplementary Table S8**). Drought stress significantly up-regulated the expression of hexokinase (HK), sucrose-phosphate synthase (E2.4.1.14), trehalose-6-phosphate phosphatase (otsB), sucrose synthase (SUS), glucan endo-1,3-beta-glucosidase (EC:3.2.1.39), alpha-amylase (AMY), bglX, endoglucanase (E3.2.1.4), granule-bound starch synthase (WAXY), ADP-sugar diphosphatase (NUDX14), and beta-amylase (BAM) genes in CK *vs* D1 and CK *vs* D2, whereas the expression of scrK, glgA, and pgm genes was down-regulated. However, in the corresponding D1 *vs* D1S and D2 *vs* D2S comparisons, these expression changes were reversed.

### 3.9 Influence of exogenous SA on photosynthesis, antenna proteins involved in photosynthesis, and genes linked to the biocarbon fixation pathway under drought stress

**Figure 11:**
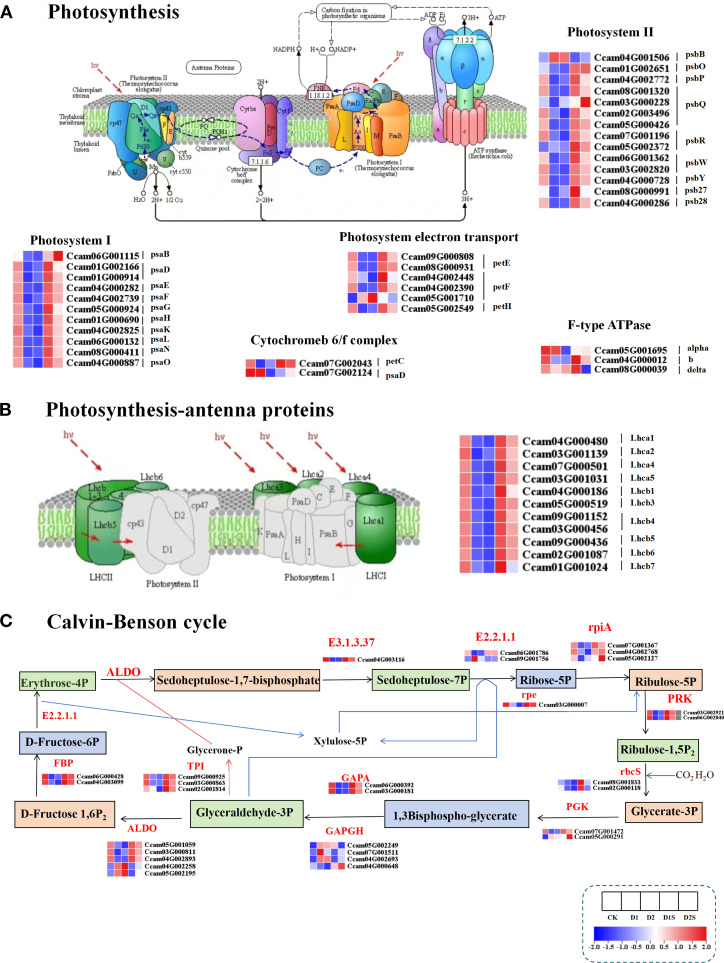
Impact of drought and SA treatment on the expression levels of differentially expressed genes (DEGs) associated with the photosynthesis pathway **(A)** Photosynthesis. **(B)** Photosynthesis-antenna proteins. **(C)** Carbon fixation in photosynthetic organisms. ALDO, fructose bisphosphate aldolase, class I; GAPDH, glyceraldehyde-3-phosphate dehydrogenase (phosphorylating); rbcS, ribulose bisphosphate carboxylase small chain; GAPA, glyceraldehyde-3-phosphate dehydrogenase (NADP+) (phosphorylating); E2.2.1.1, transketolase; PGK, phosphoglycerate kinase; FBP, fructose 1,6-bisphosphatase I; PRK, phosphoribulokinase; E3.1.3.37, sedoheptulose bisphosphatase; rpiA, ribose 5-phosphate isomerase A; TPI, triosephosphate isomerase (TIM); ppc, phosphoenolpyruvate carboxylase.

This study examines the effects of exogenous SA on photosynthesis in *C. camphora* by analyzing the expression of 36 DEGs associated with the photosynthetic pathway under drought conditions (**Figure 11A**; **Supplementary Table S9**). There are 11 DEGs associated with photosystem I, 14 DEGs related to the photosystem II complex, 3 DEGs for the cytochrome b6-f complex, 6 DEGs involved in the photosystem electron transport pathway, and 2 DEGs about f-type ATPase. The study results revealed gene expression levels associated with photosystem I, photosystem II, cytochrome b6-f complex, photoelectron transfer chain, and F-type ATPase were significantly down-regulated in the CK *vs* D1 and CK *vs* D2 groups. In contrast, comparisons of D1 *vs* D1S and D2 *vs* D2S revealed that, aside from the two genes psbB and delta, which exhibited down-regulation, the remaining related genes demonstrated a significant up-regulation trend.

Furthermore, experimental data indicated that the application of exogenous SA markedly enhanced the expression of genes associated with photosynthetic antenna proteins (**Figure 11B**; **Supplementary Table S10**). A total of 10 DEGs were identified, comprising Lhca1/2/4/5 and Lhcb1/3/4/5/6/7. These genes exhibited a significant up-regulation in both the D1 *vs* D1S and D2 *vs* D2S comparison groups, while demonstrating a notable down-regulation in the CK *vs* D1 and CK *vs* D2 groups. This suggests that SA may enhance photosynthesis in *C. camphora* under drought conditions by increasing light energy acquisition. Meanwhile, the study identified 44 DEGs related to photosynthetic biocarbon fixation (**Figure 11C**; **Supplementary Table S10**), with 30 of these genes participating in the Calvin cycle process within vascular bundle sheath cells. Specifically, the phosphoribulokinase (PRK), triosephosphate isomerase (TIM) (TPI), ribulose-bisphosphate carboxylase small chain(rbcS), phosphoglycerate kinase (PGK), sedoheptulose bisphosphatase (E3.1.3.37), fructose-1,6-bisphosphatase I(FBP), sedoheptulose bisphosphatase (rpiA), glyceraldehyde-3-phosphate dehydrogenase(NADP+)(phosphorylating)(GAPA)and ribulose phosphate 3-epimerase (rpe) genes exhibited up-regulation in the D1*vs*D1S and D2*vs*D2S comparator groups, while demonstrating down-regulation in the CK*vs*D1 and CK*vs*D2 groups. Conversely, the GAPDH and ALDO genes showed mixed patterns of up- and down-regulation.

### 3.10 Transcription factor analyses

**Figure 12:**
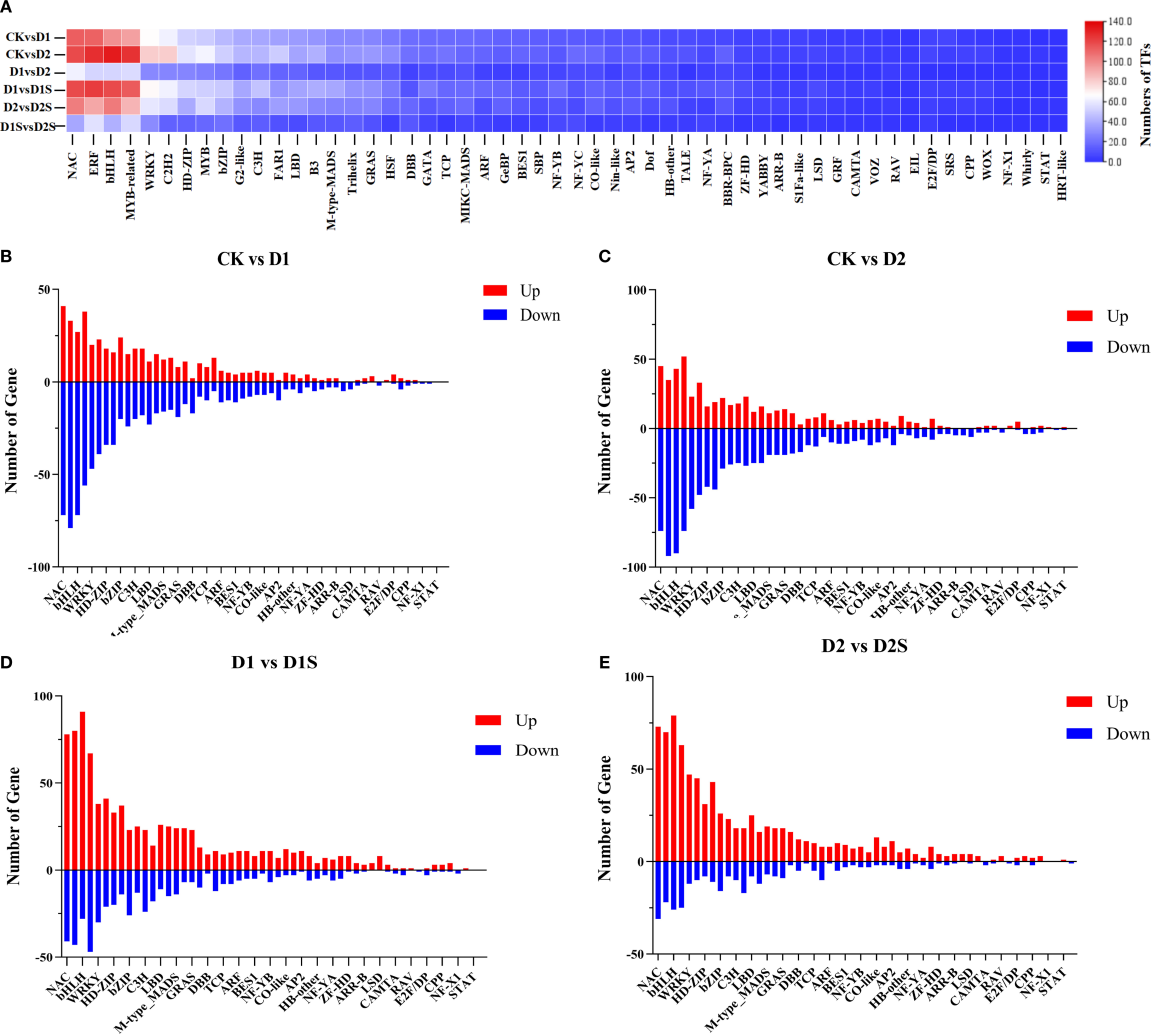
Analysis of differential transcription factors under drought stress and SA treatment. **(A)**. Heat map of TF families in various comparisons. **(B–E)**. Analysis of associated TFs in various treatment groups for CK versus D1, CK versus D2, D1 versus D1S, and D2 versus D2S, respectively. In each bar graph, genes that are up-regulated are indicated in red, while those that are down-regulated are denoted in blue.

Transcription factors (TFs) serve a pivotal regulatory function in plants when confronted with adverse conditions. These identified transcription factors mainly belong to 54 transcription families, including NAC ERF、bHLH、MYB_relate、WRKY、C2H2、HD-ZIP、MYB、bZIP、G2-like、C3H、FAR1、LBD、B3、M-type_MADS、Trihelix、GRAS、HSF、DBB、GATA、TCP、MIKC_MADS、ARF、GeBP、BES1、SBP、NF-YB、NF-YC、CO-like、Nin-like、AP2、Dof、HB-other、TALE、NF-YA、BBR-BPC、ZF-HD、YABBY、ARR-B、S1Fa-like、LSD、GRF、CAMTA、VOZ、RAV、EIL、E2F/DP、SRS、CPP、WOX、NF-X1、Whirly、STAT、HRT-like (**Figure 12A**; **Supplementary Table S12**). 1265, 1512, 603, 1393, 1146, and 478 differentially expressed TFs were handsomely selected in CK*vs*D1, CK*vs*D2, D1 *vs* D2, D1 *vs* D2, D1 *vs* D1S, D2 *vs* D2S, and D1S *vs* D2S, respectively. NAC, bHLH, ERF, and MYB-related families had the most differentially expressed TFs among CK*vs*D1, CK*vs*D2, D1 *vs* D1S, and D2 *vs* D2S, based on gene counts. CK *vs* D1 has 113, 112, 99, and 94 (**Figure 12B**). Ck*vs*D2 was 119, 127, 133, and 126 (**Figure 12C**). D1 *vs* D1S has 119, 123, 119, and 114 (**Figure 12D**). D2*vs*D2S has 104, 92, 105, and 88 (**Figure 12E**).

**Figure 13:**
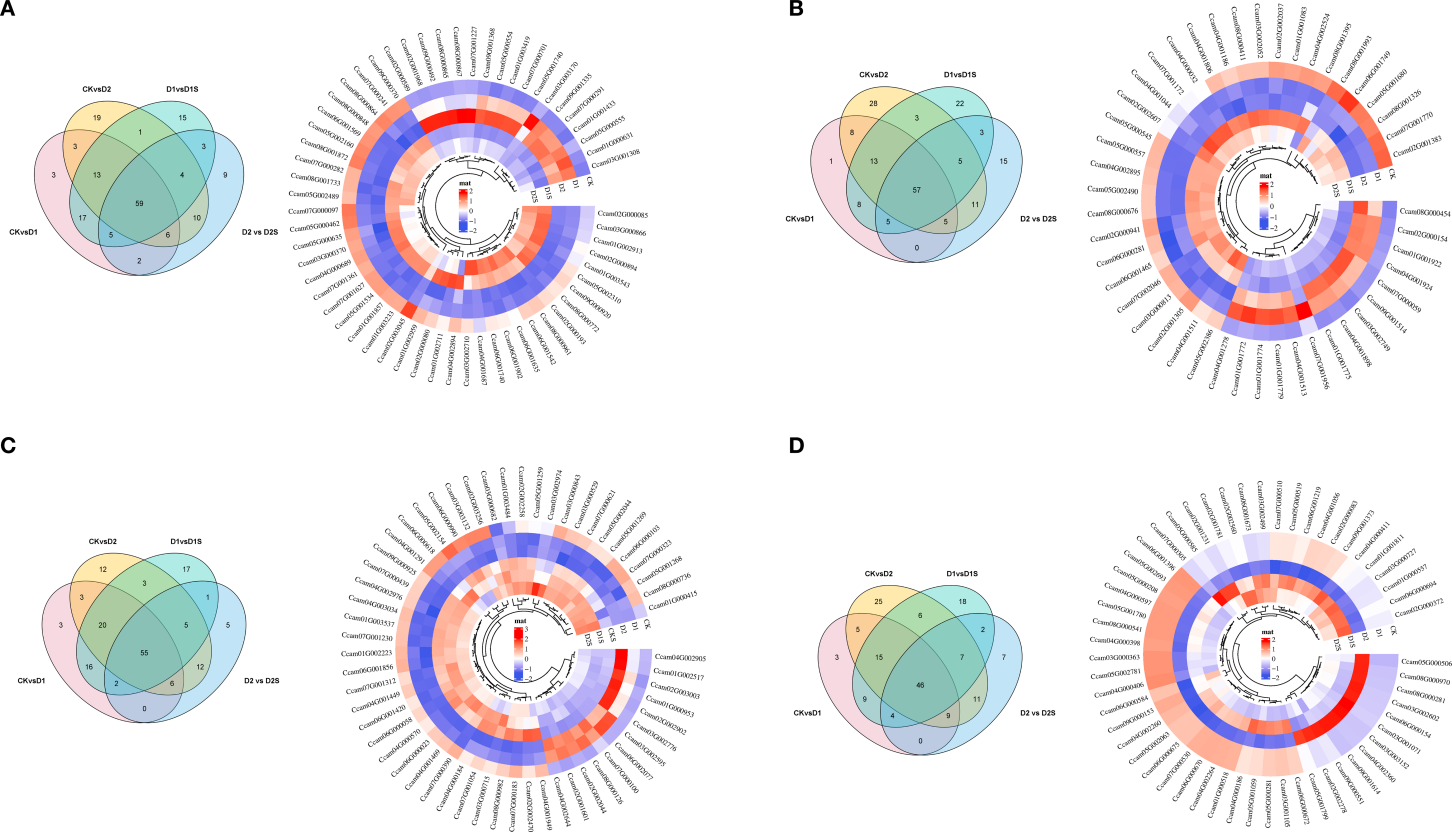
**(A–D)** Wayne plots and expression profiles of shared DEGs for NAC, bHLH, ERF, and MYB-related genes in the four comparison groups, respectively. FPKM-based differential gene heatmap.

We also performed Venn diagram and cluster heatmap analyses on these four comparison groups’ top four transcription factors (**Figure 13**; **Supplementary Table S13**). The study identified 60, 60, 59, and 48 transcription factors across the four groups. It was observed that the majority of genes were repressed under drought stress, while a significant increase occurred following the application of SA. In the phytohormone pathway, 37 transcription factors were found, including bHLH (10), bZIP (6), LBD (5), and another bHLH (4) family. During this starch/sucrose metabolic route, 38 transcription factors were found, including C2H2 (7), HD-ZIP (4), and ERF (4). Additionally, transcription factors were found in phenylalanine synthesis (14), photosynthesis (6), photosynthetic antenna proteins (3), and carbon fixation (18) (**Supplementary Table S14**). Thus, transcription factors may modulate metabolic processes and phytohormone signaling pathways in drought stress responses.

### 3.11 qRT-PCR validation of transcriptomics data

**Figure 14:**
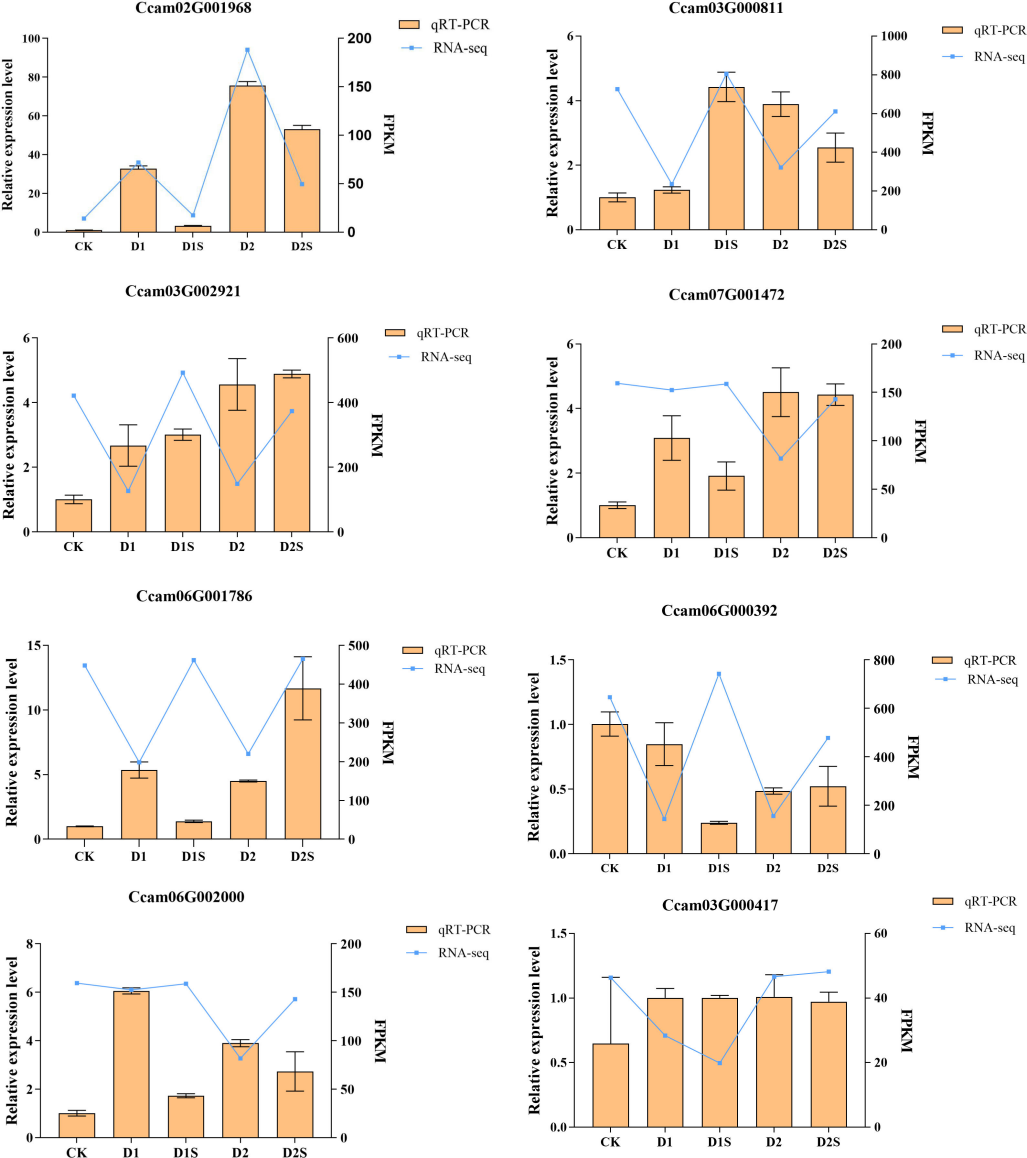
Presents the findings from the qRT-PCR analysis of 8 DGEs. The blue folded line in the figure illustrates the outcomes of the correlation analysis conducted between RNA sequencing data and qRT-PCR expression profiles.

In order to ascertain the reliability of RNA-Seq data, this study selected seven key genes of *C. camphora* for validation through real-time fluorescence quantitative PCR, as illustrated in **Figure 14**. The experimental results indicated that the gene expression profiles identified by qRT-PCR were largely consistent with the expression trends observed in the RNA-Seq sequencing data, thereby confirming the accuracy of the transcriptome sequencing results.

## 4 Discussion

**Figure 15:**
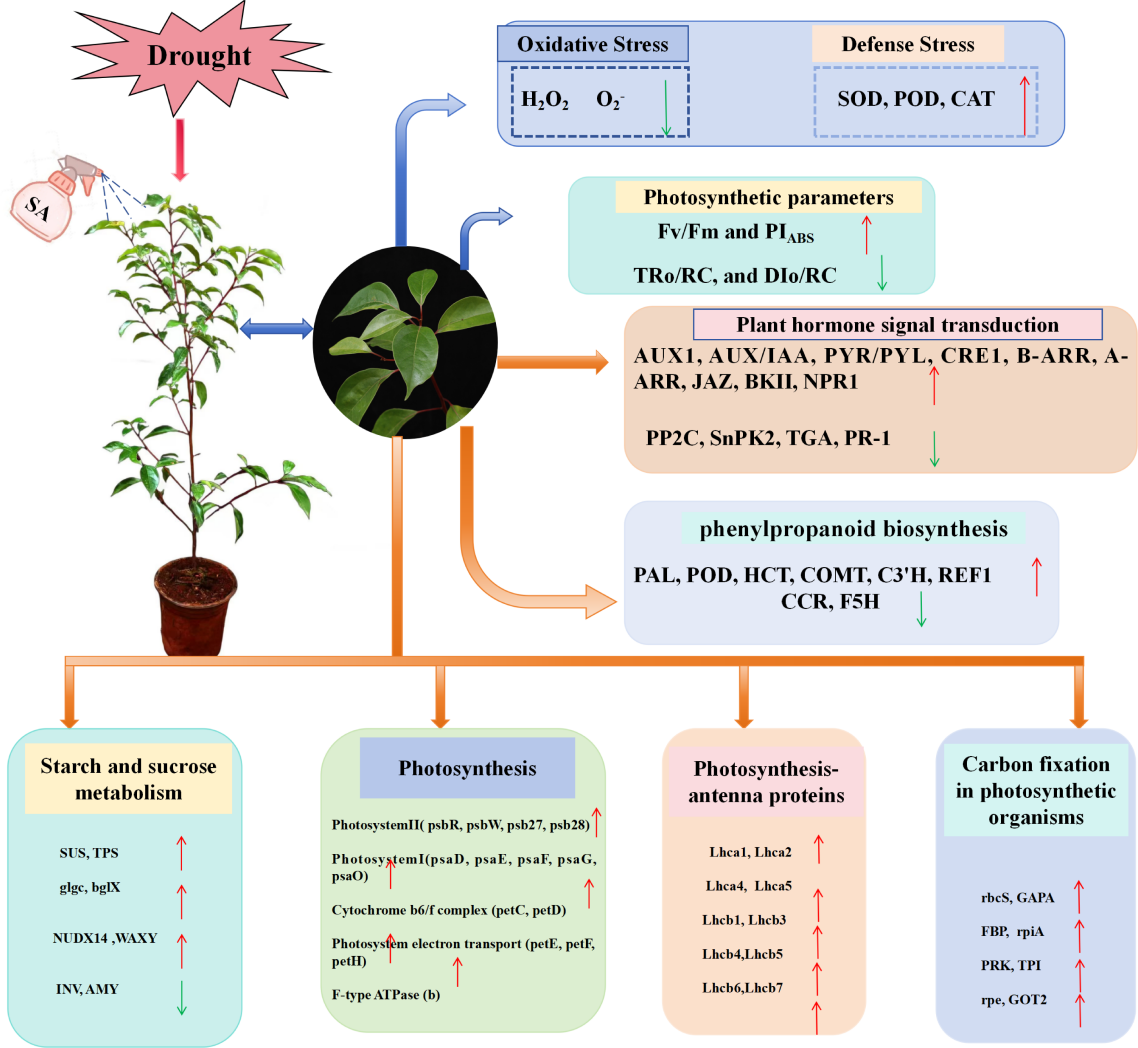
Summary of the response pattern of SA treatment on *C. camphora* seedlings under drought stress.

The effects of drought profoundly influence trees by impacting several facets of their physiology, development, production, and nutritional status (Oguz et al., 2022; Zahedi et al., 2024). This underscores the importance of implementing effective drought mitigation strategies to enhance plant drought tolerance and promote healthy growth. Salicylic acid, a crucial endogenous hormone in plants, plays a significant role in the stress resistance response through various physiological mechanisms. The specific mechanism by which SA improves drought tolerance in plants remains inadequately understood. This study integrated physiological index measurements with transcriptomic analyses to systematically examine the response and tolerance mechanisms of *C. camphora* seedlings under drought stress from multiple perspectives (**Figure 15**).

### 4.1 The application of exogenous salicylic acid enhances physiological parameters in *C. camphora* seedlings during drought stress

The most intuitive impact of drought stress on plants is growth inhibition, or even death (Zeng et al., 2022). Drought leads to notable phenotypic alterations, such as wilting of leaves, a decline or even yellowing of leaf coloration, and the drying and curling of leaf margins (Gai et al., 2020; Sun et al., 2022). Compared with normal conditions, drought stress inhibits the growth of *C. camphora* seedlings, such as leaf wilting and chlorosis (**Figure 1**). Exogenous SA can augment the drought resilience of plants. The application of exogenous SA during drought stress can markedly reduce leaf wilting signs in young camphor tree seedlings. Wheat exhibited similar results, with 50 μM SA spraying enhancing its drought resistance (Khalvandi et al., 2021).

Changes in PSI, PSII, and their photoelectron transfer chain in various plants are reflected in the kinetics of chlorophyll fluorescence (Guo et al., 2016). Moreover, the parameter PIABS, employed for an extensive evaluation of PSII’s reaction to environmental conditions, diminished markedly with escalating drought intensity, indicating a decline in PSII activity (Wang et al., 2025). Fv/Fm and PI_abs_ decrease significantly in *C. camphora* seedlings under drought stress. During periods of drought stress, both ABS/RC and TRO/RC initially exhibited an increase, followed by a subsequent decrease as the duration of stress prolonged, while ETO/RC showed a decline. Nonetheless, as a result of the reduction in light energy conversion, there is an increase in the associated DIO/RC. Notably, in this study, the exogenous administration of SA significantly enhanced the Fv/Fm ratio and PIabs level, while diminishing the photosynthetic energy indices ABS/RC, TRo/RC, and DIo/RC. Consequently, we assert that SA may alleviate the detrimental effects of drought stress on the photosynthetic apparatus, thus enhancing light energy conversion efficiency and photosystem activity (Gao et al., 2023; Moustakas et al., 2023).

The accumulation of ROS is considered a symptom of the stress response in plants when subjected to drought. The levels of malondialdehyde (MDA), H_2_O_2_, and O_2_^−^, which are induced by oxidative stress, can reflect the ROS status of plants (Wang et al., 2023). This study found that H_2_O_2_ and O_2_^−^ levels increased during drought stress, whereas a decrease was noted after SA treatment. This suggests that drought stress can induce the generation of reactive ROS and modify cell membrane structure, resulting in alterations in lipid peroxidation inside the membranes. Nonetheless, SA can mitigate the harm inflicted by stress to a degree (Huang et al., 2022). Numerous studies have indicated that under drought stress, the activity of antioxidant enzymes (including SOD, POD, and CAT) is elevated, which is thought to be associated with increased production of ROS (Wang et al., 2025). This study found that the activities of SOD, POD, and CAT increased during drought stress, but SA treatment greatly lowered the activities of these enzymes. This aligns with the findings of Huang et al. (2022), suggesting that the synergistic effects of these enzymes are essential for neutralizing reactive oxygen species and mitigating oxidative stress within the plant’s antioxidant defense system (Saheri et al., 2020; Rajput et al., 2021). Notably, exogenous SA therapy proved more efficacious for short-term stress and mild drought compared to long-term stress and severe drought. Consequently, we posit that SA may directly or indirectly stimulate antioxidant enzyme activity, diminish ROS accumulation, mitigate oxidative stress in young *C. camphora* seedlings under drought stress, and consequently improve their photosynthetic efficiency and overall growth status (La et al., 2019; Elsisi et al., 2024).

### 4.2 Exogenous SA regulates phytohormone signaling pathways under drought stress

Plant hormones are capable of meticulously regulating the growth, development, and physiological processes of plants through the modulation of their concentration levels (Kumari et al., 2023).

Auxin can profoundly influence plant drought tolerance as well as growth and development (Yu et al., 2022; Guan et al., 2023). This study revealed that drought stress resulted in the downregulation of Aux/IAA and AUX1 gene expression, but treatment with SA led to their upregulation. The expression of ARF, GH3, and SAUR genes exhibited a heterogeneous pattern of upregulation and downregulation, reflecting variations in their responses. Notably, the GH3.5 enzyme targets IAA and SA, signifying their involvement in the interaction between these IAA and SA. In Arabidopsis, AtGH3-5/WES1 augments plant resilience to thermal and drought stress by engaging in the SA/ABA-mediated plant defense response system (Park et al., 2007; Bao et al., 2024). Furthermore, ROS is essential for preserving stem cell properties and the spatial distribution of auxin, whereas SA modulates ROS levels by regulating the expression of RBOHs (Rawat and Laxmi, 2025). Consequently, SA-mediated drought tolerance in *C. camphora* may be associated with the expression of auxin biosynthesis genes and reactive oxygen species control (Shi et al., 2024b; Rawat and Laxmi, 2025).

ABA serves as a pivotal signaling hormone during drought stress. Under normal circumstances, ABA swiftly accumulates and connects with ABA receptors to create PYR/PYL/RCAR-ABA-PP2C complexes. This process liberates SnRK2 kinases from the inhibition of PP2C phosphatases, enabling SnRK2s to phosphorylate and activate downstream target proteins, thus facilitating stomatal closure in response to drought stress (Hsu et al., 2021; Zhou et al., 2025). Research demonstrates that ABA and SA display both synergistic and antagonistic interactions (Hsu et al., 2021; Zhou et al., 2025). Furthermore, during drought stress, salicylic acid therapy diminishes the expression of the abscisic acid synthesis-related gene NCED3. It has been indicated that SA signaling in guard cells is facilitated by its receptor NPR1. During drought stress, SA and ABA signals are integrated in guard cells through the CBK pathway, with SA activating and integrating into the Ca^2+^/CPK-dependent branch of the ABA signaling pathway via the peroxidase-mediated reactive oxygen species signaling pathway (Prodhan et al., 2018; Li et al., 2023; Elsisi et al., 2024). This study revealed that the ABA signaling pathway was upregulated in the majority of genes under drought stress, whereas PYR/PYL gene expression was downregulated. The administration of salicylic acid resulted in the upregulation of PYR/PYL expression and the downregulation of PP2C and SnRK2 expression. We hypothesize that SA-induced drought tolerance in *C. camphora* may be facilitated by the expression of genes associated with the ABA signaling pathway and the regulation of ROS, thereby establishing a stable regulatory circuit that collaboratively governs stomatal closure and stress responses.

Comprehensive studies demonstrate that although the jasmonic acid (JA) and salicylic acid (SA) signaling pathways are typically antagonistic, changes in the content of one hormone may trigger similar changes in the content of the other, thus enabling both to play the same role in inducing plant defense gene expression and regulating defense responses (Wang et al., 2021; Mu et al., 2025). Furthermore, prior research has elucidated the mechanisms governing the interaction between the JA and SA signaling pathways, identifying multiple genes, including MYC2, ERF1, MAPK (Mitogen-Activated Protein Kinase), GRX480(Glutaredoxin 480), NPR1, WRKY62, WRKY70, PDF 1.2(Plant Defensin 1.2), TGAs, ORA59(Oleate-Responsive AP2/ERF 59), and JAZ, that mediate the antagonistic relationship between SA and JA (Mu et al., 2025; Zhang et al., 2025a). Notably, the existence of NPR1 orthologs in the common ancestor of terrestrial plants indicates that interactions between the jasmonic acid and salicylic acid signaling pathways may be prevalent across the plant kingdom (Thaler et al., 2012). This study demonstrated that exogenous SA treatment significantly enhanced the expression levels of JAZ and MYC2 genes, suggesting an interaction between SA and JA, and that the JA signaling pathway is crucial for the improvement of drought alleviation in *C. camphora* by SA (Zhang et al., 2025a).

In addition, SA, GA, ABA, and MeJA functioned as inducers for the transcription factor TGA, which played a crucial role in regulating disease resistance and stress-related signaling pathways, consequently improving plant stress tolerance (Chen et al., 2021; Shi et al., 2024b). NPR1 is a crucial regulator that mediates both biotic and abiotic stress in plants (Park et al., 2021; Jia et al., 2023). The induction of PR-1 gene expression by SA in tomato under drought conditions demonstrates that the PR gene is induced in response to both biotic and abiotic stresses (Akbudak et al., 2020). We discovered that NPR1 expression was up-regulated while TGA and PR-1 were up-regulated during drought stress, but exogenous SA treatment demonstrated the opposite trend. It is hypothesized that the administration of exogenous salicylic acid enhances the production of endogenous salicylic acid in plants, but upon reaching a certain level, it subsequently suppresses salicylic acid synthesis. This regulatory action may be due to a balanced SA signaling process, where plants actively inhibit the signaling process upon excessive SA accumulation to prevent over-immunization (Miura et al., 2013; Niu et al., 2023; Zhang et al., 2025a). Therefore, research indicates that the presence of intricate interactions within phytohormone signaling establishes a dynamic regulatory network characterized by signal cross-talks (Salvi et al., 2021; Elsisi et al., 2024) (**Table 1**).

**Table 1:** Regulatory mechanisms of genes in salicylic acid-induced physiological processes under drought stress.

Gene	Regulatory mechanism	Physiological effects	References
NPR1	NPR1 (nonexpresser of pathogenesis-related gene 1), SA receptor/transcriptional co-activator; SA activates the NPR1 signaling pathway and regulates the expression of defense gene PR through TGA transcription factors	Initiate the systemic acquired resistance (SAR) and augment antioxidant properties.	(Zavaliev and Dong, 2024)
PR1	PR(pathogenesis-related, used as markers for SA-mediated activation of SAR (systemic acquired resistance)	antifungal activity, degradation of cell wall macromolecules	(Zhang et al., 2025a)
PAL	Phenylalanine ammonia-lyase, conversion of phenylalanine to trans-cinnamic acid	Facilitats SA biosynthesis	(Huang et al., 2010)
AIM	Beta-oxidation multifunctional protein. Conversion of trans-cinnamic acid to benzoic acid	SA regulates stomatal aperture through the OsWRKY45-reactive oxygen pathway	(Xu et al., 2023a)
P5CS	pyrroline-5-carboxylate synthase, SA promotes proline synthesis by inducing P5CS gene expression	Maintain cell turgor pressure and enhance water retention capacity	(La et al., 2019)
TRXh5/GRXC9	Thioredoxin h5/Glutaredoxin C9, SA regulates the expression of redox regulatory genes (TRXh5 and GRXC9)	Regulation of redox signals	(La et al., 2019)
POD/SOD/CAT/APX/GPX	Peroxidase (POD)/Superoxide dismutase (SOD)/Catalase (CAT)/Ascorbate peroxidase (APX)/Glutathione peroxidase (GPX). These enzymes enhance the activity of antioxidant enzymes and scavenge reactive oxygen species (ROS).	Antioxidant defense system, maintaining ROS dynamic balance	(Elsisi et al., 2024)
NCED	9-cis-epoxycarotenoid dioxygenase, which catalyzes the oxidative cleavage of the epoxide carotenoid 9-cis isomer, converting 9-cis-meso-zeaxanthin and 9′-cis-neoxanthin into xanthoxin	promote ABA synthesis.	(Zhang et al., 2025a)

### 4.3 Exogenous SA regulates phenylpropanoid pathways under drought stress

The metabolism of phenylpropanoids, recognized as a highly significant metabolic pathway in plants, plays an essential role in plant growth and development, adaptation to environmental conditions, and adaptability to stressors (Zhang and Liu, 2015; Dong and Lin, 2021). PAL, C4H, and 4CL are three primary enzymes that play a significant role in the metabolic pathway of phenylalanine (Zhang and Liu, 2015). Lignans and flavonoids, as significant metabolites within the phenylpropanoid biosynthesis pathway, are crucial for plant growth and development, and they additionally contribute to the regulation of drought tolerance (Yan et al., 2021; Li et al., 2022). Under conditions of drought stress, the accumulation of lignin results in the thickening of the cell wall, which diminishes the osmotic capacity of the cell, thereby allowing the cell to continue expanding despite the lack of water (Xu et al., 2022).

Several genes associated with lignin biosynthesis have been identified, such as 4CL, CCR, CAD, COMT, CCoAOMT, and HCT. The expression levels of these genes can influence the accumulation of lignin (Liu et al., 2018; Shi et al., 2024b). In our investigation, the expression of PAL, CAD, 4CL, COMT, and CCoAOMT genes was found to be repressed under conditions of drought stress, while the application of SA resulted in the activation of these genes. Furthermore, HCT, characterized by its dual catalytic functions, is primarily engaged in the synthesis of lignin, while F5H is significant in the biosynthetic pathway of phenylpropanoids, contributing to cell wall enhancement and substance transport (Dong and Lin, 2021; Hu et al., 2023). The expression levels of HCT and F5H genes exhibited a down-regulation in response to drought stress; however, treatment with SA resulted in an up-regulation of HCT expression. Exogenous SA can directly or indirectly stimulate the production of secondary metabolites. It is thus hypothesized that SA may stimulate the production of lignin and phenolic compounds, fortify cell walls, diminish water loss, and elevate the expression of associated genes, consequently activating the antioxidant system to some degree and augmenting plants’ drought resistance (Liu et al., 2023b; Zhang et al., 2023b).

### 4.4 Exogenous SA regulates starch and sucrose metabolic pathways under drought stress

The metabolism of starch and sucrose is integral to the processes of carbon metabolism (Lu et al., 2019; Gai et al., 2020). Research indicates that drought and salt stress negatively impact the metabolic pathways associated with starch and sucrose, primarily by inhibiting photosynthesis and influencing gene expression, enzyme activities, and carbohydrate accumulation (Li et al., 2024). In instances of drought, when photosynthesis is constrained, a plant will undergo the process of re-decomposing starch to provide supplementary energy and carbon (Gai et al., 2020). Several research investigations have demonstrated that the process of starch synthesis encompasses a range of amylases, predominantly including sucrose synthase (SS), ADP glucose pyrophosphorylase (AGPase), granule-bound starch synthase (GBSS), starch branching enzyme (SBE), and debranching enzyme (DBE) (Lu et al., 2019).

Moreover, sucrose, being a principal product of photosynthesis in higher plants, holds significant importance in plant signaling and adaptation to adverse conditions (Sun et al., 2024). Sucrose phosphate synthase (SPS) plays a critical role in the biosynthesis of sucrose, catalyzing the conversion of fructose-6-phosphate and uridine diphosphate glucose (UDPG) into sucrose-6-phosphate (Du et al., 2020; Xu et al., 2022). Whereas sucrose synthase (SUS) and invertase (INV) serve as critical enzymes in sucrose degradation, facilitating the hydrolysis of sucrose to hexose (Xu et al., 2022). Reports indicate that the equilibrium between starch and sucrose metabolism is upheld through the down-regulation of INV inhibitors, which serves to decrease INV activity (Thomas et al., 2022). Additional research has demonstrated that the expression of genes associated with starch synthesis, such as AGPase and SS, was inhibited, while the expression of genes related to starch degradation, including AMY and BAM, was activated. Furthermore, there was a notable increase in the expression of SuSy genes under conditions of water stress (Liu et al., 2016; Gai et al., 2020). This study found that the application of SA promoted sucrose synthesis through the up-regulation of the SuS gene expression. Conversely, the AMY and INV genes associated with the degradation of starch and sucrose exhibited down-regulation. It was posited that SA may stimulate a metabolic transition towards storage in *C. camphora* seedlings experiencing drought stress, consequently improving their drought tolerance.

### 4.5 Exogenous SA regulates the photosynthetic pathway under drought stress

Photosynthesis represents the initial series of processes influenced by drought stress. Under conditions of drought, alterations in cell expansion pressure impact the arrangement of stomata, with stomatal closure serving as the primary response of the plant (Sharma et al., 2020). This response results in a reduction of net CO_2_ assimilation, which ultimately has repercussions for photosynthesis. Research indicates that SA can significantly improve photosynthesis in plants when subjected to environmental stresses (Cheng et al., 2020; Shi et al., 2024a). The PSII core complex proteins Psb27, Psb28, and PsbW are essential for photodamage repair processes and maintaining PSII stability (Sakata et al., 2013; Wang et al., 2024). Studies revealed that the expression of these proteins was upregulated in response to SA treatment and downregulated under conditions of water stress. Moreover, PsbR, a key component of PSII, improves oxygen release by optimizing electron transport and water oxidation (Liu et al., 2023a). PsaD and PsaE play crucial roles in the electron transport chain of PSI, facilitating electron transfer and the binding of oxygen-reduction proteins to NADP+ oxidase, while PsaF is involved in light-harvesting protein assembly and interaction (Xu et al., 2023b). The treatment with SA resulted in a notable increase in the expression levels of PSI-related genes, including PsaD, PsaE, and PsaF. This observation indicates that SA may play a role in improving the structural stability and functional activity of the photosynthetic system through the synergistic regulation of various functional genes associated with PSI and PSII.

In conditions of drought, the diminished efficiency of electron transfer during photosynthesis results in an obstruction of ATP synthesis, consequently imposing constraints on metabolic processes (Singh et al., 2014). The cytochrome b6f complex establishes the gradient of protons across the thylakoid membrane and works in conjunction with other proton gradients to facilitate ATP synthesis, thereby supplying energy for carbon fixation in plants (Wang et al., 2024). SA has the potential to enhance the expression of genes like the F-type ATPase, thereby contributing to enhanced photosynthetic capacity and energy supply under conditions of drought stress (Moustakas et al., 2023). Furthermore, under short-term drought, LHCII separates from the PSII supercomplex, while under long-term drought, the PSII core is degraded (Li et al., 2023; Sun et al., 2024). LHC-related gene expression was down-regulated under drought stress, whereas SA treatment significantly up-regulated it. Consequently, we proposed that SA could preserve the integrity of the photosynthetic apparatus through the regulation of LHC gene transcript levels, which may enhance the light energy capture capacity of *C. camphora* during drought stress, mitigate the phenomenon of drought-induced photoinhibition, and warrant further investigation into the underlying mechanisms.

The Calvin cycle (or C3 reaction cycle) serves as the main mechanism for carbon fixation during the process of photosynthesis in plants. This cycle encompasses three fundamental stages: carboxylation, reduction, and regeneration (Chen et al., 2022; Wang et al., 2022). This research screened 29 DEGs linked to the Calvin cycle, including genes encoding major enzymes, including rbcS, PGK, GAPA, FBP, rpiA, PRK, TIM, and rpe, whose expression was down-regulated during drought. This research demonstrates that the external application of SA during periods of drought stress significantly elevated the activities of crucial enzymes involved in different phases of the Calvin cycle. This enhancement contributed to an improved photosynthetic capacity and increased drought tolerance in *C. camphora* by facilitating the processes of photosynthetic electron transfer and phosphorylation under drought conditions.

## 5 Conclusions

The research studied the effects of exogenous SA on the physiological and transcriptomic responses of *C. camphora* seedlings under drought conditions, uncovering various signaling pathways through which SA influences plant reactions to drought stress. Results indicated that SA could enhance photosynthesis in *C. camphora* seedlings and significantly mitigate the negative impacts of drought stress. Concerning antioxidant defenses, treatment with SA resulted in an increase in the activity of antioxidant enzymes and a reduction in ROS levels, thereby maintaining intracellular stability and effectively mitigating oxidative damage to membranes. Moreover, transcriptome analysis indicated that SA alleviated drought stress damage and improved drought resistance in *C. camphora* seedlings via modulating phytohormone signaling pathways, phenylpropanoid metabolism, the metabolism of starch and sucrose, and photosynthesis-related genes and transcription factors.

## Data Availability

The datasets presented in this study can be found in online repositories. The names of the repository/repositories and accession number(s) can be found in the article/**Supplementary Material**.
